# CD8 T lymphocytes deploy embryonic cell cycle control mechanisms for rapid cell proliferation

**DOI:** 10.1038/s44319-026-00830-4

**Published:** 2026-06-18

**Authors:** David A Lewis, Ananya Kar, Alice Savage, Van Kelly, David Wright, Devin Tan, Mary Tozer, Christina Rollings, Doreen A Cantrell, Rose Zamoyska, Tony Ly

**Affiliations:** 1 Institute of Immunology and Infection Research, University of Edinburgh, Ashworth Laboratories, Edinburgh, UK; 2 Wellcome Centre for Cell Biology, University of Edinburgh, Edinburgh, UK; 3 Molecular, Cell and Developmental Biology, School of Life Sciences, University of Dundee, Dundee, UK; 4 Cell Signalling and Immunology, School of Life Sciences, University of Dundee, Dundee, UK

**Keywords:** Cell Cycle, Immunology, Stem Cells & Regenerative Medicine

## Abstract

Rapid proliferation of CD8 T cells is crucial for adaptive immunity against viral infection. CD8 T cells can complete division cycles in less than 6 h, representing a physiological extreme for somatic mammalian cells. Embryonic stem cells utilize specialized cell cycle control mechanisms, including subdued periodic expression, for rapid cell division cycles. CD8 T cell cycle control remains poorly understood. Here, we test whether CD8 T cells utilize embryonic mechanisms to promote rapid cell cycles. We comprehensively measure protein abundances in G1, S, and G2&M phases in three murine cell types: CD8 T cells, embryonic stem cells, and fibroblasts. We discover striking similarities between mESC and CD8 T cells. We demonstrate that CD8 T cells express Cyclin E1 and Emi1/Fbxo5 at high levels to promote S-phase entry. Interestingly, CD8 T cells and mESCs differ in the frequency of G2&M phase cells, the abundance of DNA replication origin licensing and initiation factors, and the abundance of APC/C substrates. Thus, somatic T cells have both unique and shared cell cycle control mechanisms to promote rapid cell cycles.

## Synopsis

CD8 T cell cycles are extremely fast for somatic mammalian cells, enabling them to quickly expand cell numbers in response to infection. Somatic T cells have both unique and shared cell cycle control mechanisms to promote rapid cell cycles.

- Cell cycle regulation is characterized using advanced proteomics in slow and fast dividing murine cell types.
- Cell cycle control mechanisms are altered in fast versus slow cycling cells.
- Fast dividing cells have constitutive expression of Cyclin E protein and high abundance of APC/C substrates.
- Cyclin E and the APC/C inhibitor Emi1 are key regulators of cell cycle speed.

## Abstract (web summary)

CD8 T cell cycles are extremely fast for somatic mammalian cells, enabling them to quickly expand cell numbers in response to infection. Somatic T cells have both unique and shared cell cycle control mechanisms to promote rapid cell cycles.

## Graphical abstract

**Figure:**
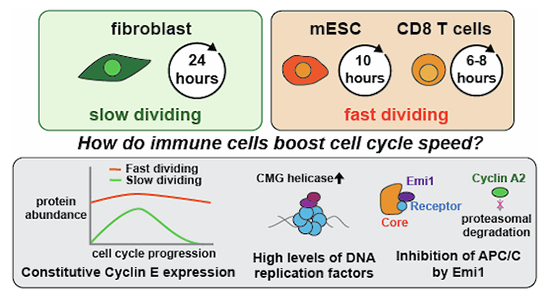

**Figure:**
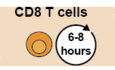

## Introduction

The structure and control of cell division cycles can differ between cell types in multicellular organisms to coordinate with developmental stage and the function of the cell type (Padgett and Santos, 2020; Li et al, 2012; Ballabeni et al, 2011; White et al, 2005; Fujii-Yamamoto et al, 2005a; Fluckiger et al, 2006; Coronado et al, 2013; Savatier et al, 1996). Activated T lymphocytes exhibit an extreme in cell division speed, proliferating more rapidly than other somatic cells, such as fibroblasts. In this study, we have focused on cytotoxic CD8 T cells. Antigen-driven activation of cytotoxic CD8 T cells causes rapid clonal expansion of antigen-selective cells. After an initial phase of cell size increase in the first ~24 h, CD8 T cells can then undergo up to four cell divisions per 24 h with a mean doubling time of 7-8 h (Plambeck et al, 2022; Dowling et al, 2014). Most activated cells then differentiate into effector CD8 T cells that mediate cytotoxic killing, with a minority becoming long-lived memory CD8 T cells that are important in adaptive immunity (Kaech and Cui, 2012). In parallel, viral replication will result in increases in pathogen burden. Timely and rapid proliferation is therefore crucial to expand antigen-specific CD8 T cell numbers and mount a successful immune response to viruses and other intracellular pathogens.

Despite the importance of rapid clonal expansion in adaptive immunity, the mechanisms of T-cell proliferation are poorly understood (Lewis and Ly, 2021). It is known, for example, that the extent of cell proliferation is dependent on the integrated signal from T-cell antigen receptor activation by peptide/MHC class I complexes, co-receptor stimulation, and cytokine exposure (Marchingo et al, 2014). However, CD8 T-cell proliferation is heterogeneous. It has been shown that fast and slow dividing subpopulations co-exist in the same in vitro culture, and that cell cycle speed is only partially heritable across generations (Plambeck et al, 2022). The mechanisms that dictate cell cycle speed remain unclear. In contrast to the established model that canonical E2F genes are pro-proliferative and, in the case of E2F1, pro-apoptotic, in vitro activated E2f1/E2f2 double knockout CD8 T cells show hyperproliferation and no observable difference in apoptosis, compared to heterozygote E2f1^+/−^E2f2^+/−^ or wild-type controls (Zhu et al, 2001). Overexpression of the cell cycle inhibitor protein p21, which potently arrests epithelial cell lines (Barr et al, 2017; Spencer et al, 2013), has no significant effect on initial CD8^+^ T cell proliferation (Daszkiewicz et al, 2015). Therefore, the cell cycles of activated CD8 T cells are remarkable in their speed and heterogeneity, and the limited evidence available suggests these cells have specialized cell cycle control mechanisms compared to other somatic cell types.

Like activated CD8 T cells, embryonic stem cells (ESCs) are multipotent and capable of rapid proliferation. ESC cell divisions are characterized by a specialized cell cycle control network (Padgett and Santos, 2020; Ballabeni et al, 2011) that produces an altered expression profile for cell cycle-promoting genes, a truncated G1 phase (Fluckiger et al, 2006; Becker et al, 2006), and rapid S-phase entry. In mouse embryonic fibroblasts (MEFs), Cyclin E, Cyclin A, and other targets of the E2F transcription factors exhibit periodic expression across the cell cycle, peaking in abundance at the G1/S transition. Cyclins E/A in complex with cyclin-dependent kinase 2 (CDK2) then hyperphosphorylate the tumor suppressor retinoblastoma protein (Rb) to relieve repression of E2F and thereby promote cell cycle gene expression (Rubin et al, 2020). In contrast, in mouse ESCs (mESCs), Cyclins E/A show dampened periodicity or constitutive expression across the cell cycle (Ballabeni et al, 2011; White et al, 2005; Fujii-Yamamoto et al, 2005b; Yang et al, 2011; Stead et al, 2002), leading to constitutive hyperphosphorylation of Rb and rapid S-phase entry. Mechanistically, the levels of Cyclin A and additional cell cycle-promoting proteins are sustained in mESCs by inhibition of the key E3 ligase, the APC/C, by its protein inhibitor, Emi1 (*Fbxo5*) (Frye et al, 2013; Reimann et al, 2001). APC/C targets many substrates for proteasomal degradation (Franks et al, 2020; Pines, 2011), including the E2F targets Cyclin A, and Skp2. Emi1 is itself an E2F target, and switches from being an APC/C substrate to an APC/C pseudo-substrate inhibitor when Emi1 levels are high (Cappell et al, 2018; Miller et al, 2006). Thus, high Emi1 levels in mESCs are thought to inhibit the APC/C and stabilize proteins like Cyclin A. Constitutive expression of Cyclin A is thought to be developmentally restricted to embryonic stem cells; however, up to now, these comparisons have not included fast-dividing somatic cell types like activated CD8 T cells.

Do CD8 T cells also utilize embryonic cell cycle control mechanisms to facilitate rapid proliferation? To address this question, we performed quantitative proteomics to measure the cell cycle-regulated proteome of activated CD8 T cells, E14 mESCs, and NIH3T3 fibroblasts. We discovered that fast-dividing CD8 and mESC cells showed constitutive expression of Cyclin E, but not Cyclin A. Cyclin E1 overexpression in the slower-dividing NIH3T3 cells increased S-phase frequency, but reduced cell counts, suggesting the existence of additional important mechanisms to enable fast cell cycle speeds. Unlike mESCs and NIH3T3s, CD8 T cells had reduced levels of Cdt1 and Cdc6, factors shown to promote rapid DNA replication origin licensing and G1 progression in hESCs. Instead, CD8 T cells had high levels of Cdc45, a rate-limiting factor in DNA replication initiation. APC/C substrate abundances were higher in mESC and, to a lesser extent, CD8 T cells, consistent with reduced APC/C activity during the G1 phase of these rapidly cycling cells. Supporting a role of APC/C, we showed that abrogation of Emi1 function in CD8 T cells suppresses G1 progression.

## Results

### CD8 T cells and mESCs share a similar doubling rate and cell cycle phase frequencies

**Figure 1:**
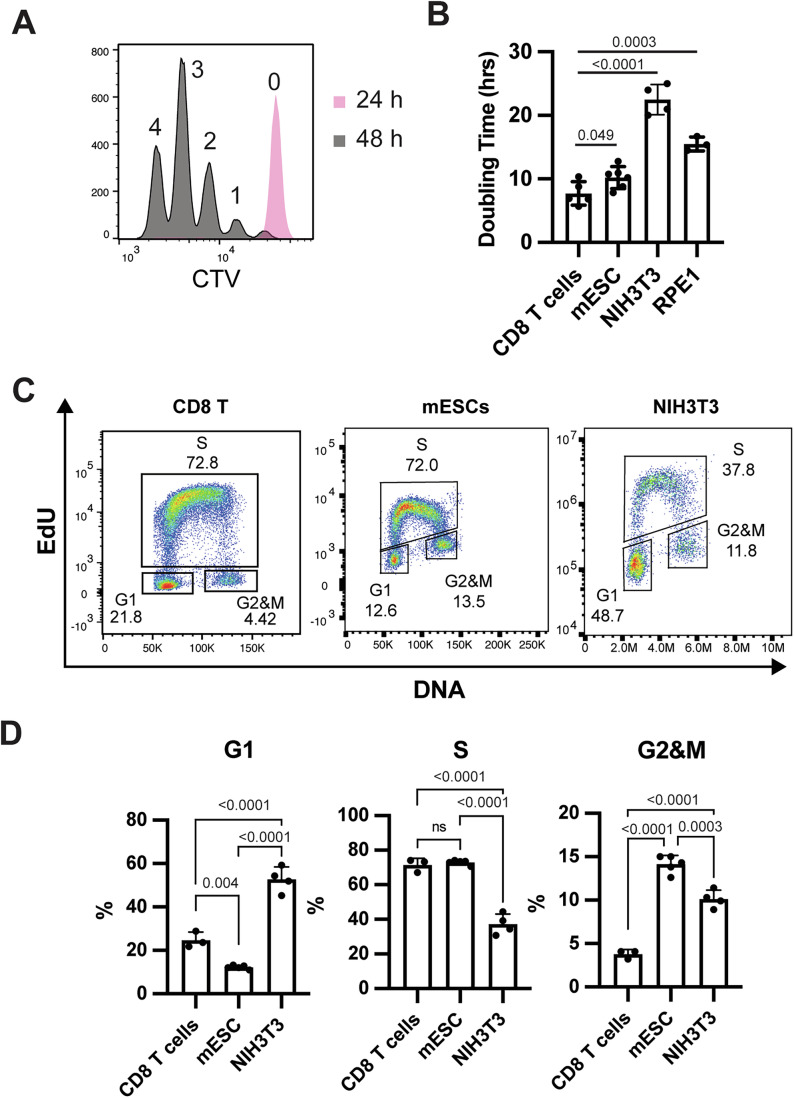
Fast CD8 T cell cycles are characterized by short gap phases. (**A**) Naive, quiescent CD8 T cells were stained with CellTraceViolet (CTV) prior to activation and were analyzed at 24 h and 48 h post activation. The number of cell divisions is indicated above the peaks. (**B**) Doubling time was estimated from daily cell counts for CD8 T cells, mouse embryonic stem cells (mESCs), NIH3T3 fibroblasts, and RPE1 cells. Results are from three to six biological replicates, error bars indicate s.e.m. (**C**) Asynchronous CD8 T cells, mESCs, and NIH3T3 cultured in an exponential growth regime were pulsed with EdU for 30 min, harvested, and subjected to Click Chemistry-based fluorescence detection. (**D**) Percentage of G1, S, and G2&M phase cells (*n* = 3-5 biological replicates), error bars indicate s.e.m. Statistical significance was determined by unpaired Student’s *t* test. Source data are available online for this figure.

We first compared murine CD8 T cell proliferation with mouse embryonic stem cells (mESCs) and mouse NIH3T3 fibroblasts. We also included in the comparison immortalized retinal pigment epithelial cells (RPE1), which is a well-characterized human cell line model used to study cell cycle control. Between 24 h and 48 h following activation, CD8 T lymphocytes underwent up to four cell divisions (Fig. 1A) and had an average cell doubling time of 7.5 h (Fig. 1B). The average cell doubling time of E14TG2a mESCs was 10 h (Fig. 1B). Both CD8 T cells and mESCs had significantly shorter doubling times than RPE1 (15 h, Fig. 1B) and the mouse NIH3T3 fibroblast cell line (22.5 h, Fig. 1B).

The difference in average cell doubling time could reflect a difference in time spent in G1. To investigate this, we used flow cytometry analysis of DNA content and EdU incorporation. The measured cell cycle phase frequencies can be used to infer phase durations (Kafri et al, 2013). This analysis showed a marked increase in S-phase cells in CD8 T lymphocytes and mESCs compared to NIH3T3 (Fig. 1C,D). This increase was primarily in early S-phase cells, which have 2N DNA content and are EdU+ (Fig. 1C). CD8 T cells showed a markedly lower frequency of cells in G2&M phase (Fig. 1D). These results suggest that mESC and CD8 T cells have a shortened G1 phase and additionally, that CD8 T cells have a shortened G2&M phase.

### Cell-type plasticity in cell-cycle-regulated protein abundance

Having established that the CD8 T cell cycle phase distribution resembled mESCs, we next investigated whether the CD8 T cell cycle control network is convergently configured to support a truncated G1 phase and rapid cell divisions.

It was previously shown that there is diminished periodicity of cell cycle-regulated (CCReg) proteins in mESCs. These proteins included key regulators such as Cyclin A2. However, conflicting results have been obtained previously, varying from no changes (White et al, 2005; Fujii-Yamamoto et al, 2005a; Savatier et al, 1996) to muted changes in CCReg protein abundances (Ballabeni et al, 2011), as recently reviewed (Padgett and Santos, 2020). These discrepancies have been ascribed to challenges in obtaining high synchrony in mESCs using conventional drug-based arrest and release approaches and the narrow linear range for immunoblot protein quantitation (Ballabeni et al, 2011).

To re-evaluate potential cell type plasticity in cell cycle regulation, and to test if CD8 T cells rely on similar adaptations to mESCs, we performed quantitative mass spectrometry-based proteomics to measure protein abundances across an unperturbed cell cycle in mouse CD8 T lymphocytes, mESC, and NIH3T3 cells. We utilized PRoteomic analysis of Intracellular iMMUnolabelled cell Subsets (PRIMMUS) (Ly et al, 2017; Kelly et al, 2022). In PRIMMUS, asynchronous cells in exponential growth are fixed in formaldehyde and immunostained for cell cycle phase-specific markers, with each cell cycle phase separately isolated by FACS for downstream proteomics sample preparation. Thus, PRIMMUS avoids potential artifacts due to cellular stresses that caused by artificial synchronization as opposed to cell cycle per se (Ly et al, 2015), and moreover, cell cycle synchronization protocols are not established for CD8 T lymphocytes. While work from our group and others has characterized mammalian cell cycle-regulated proteins previously, most of these studies were performed in human cell lines. Given that species-specific differences in cell cycle regulation between rodent and human cell lines have been described, it was crucial to compare cell types from the same species. In addition, synchronization approaches can cause differentiation in embryonic stem cells and CD8 T cells due to crosstalk between cell division and cell differentiation pathways (Pauklin and Vallier, 2013; van Haften et al, 2026; Lelliott et al, 2021; Heckler et al, 2021) that are avoided by using PRIMMUS.

**Figure 2:**
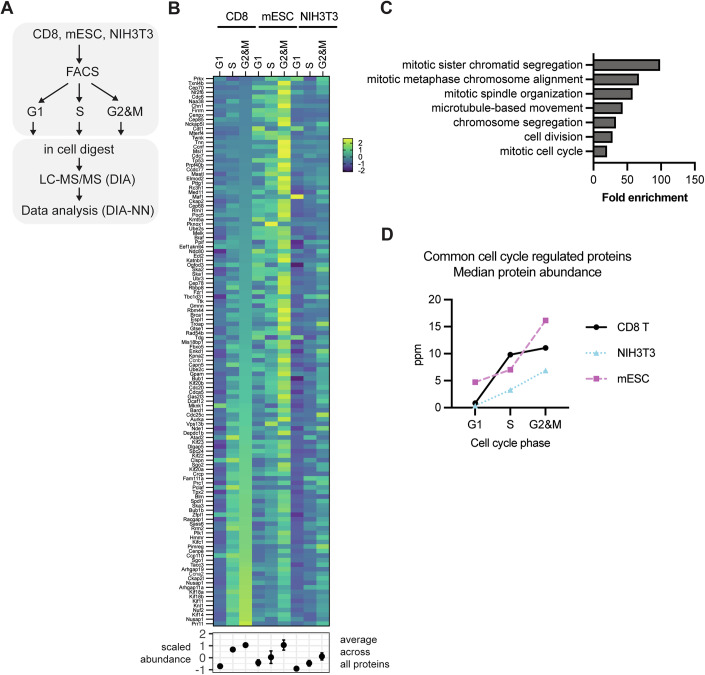
The common cell cycle-regulated protein network in CD8 T, mESC, and NIH3T3 cells. (**A**) Design of mass spectrometry-based proteomics experiment. Three mice were culled for tissue collection. CD8 T cells were expanded from dissected lymph nodes from each mouse independently for a total of three biological replicates. Three and four biological replicates were obtained, respectively, for mESC and NIH3T3, representing independent cell culture passages. (**B**) Heatmap showing the row-scaled protein abundances for the 121 proteins that are cell cycle regulated in CD8 T cells, mESC, and NIH3T3. The mean scaled abundance is shown below the heatmap. Error bars indicate standard deviation between replicates (*n* = 3–4). (**C**) GO term enrichment analysis of the common cell cycle-regulated proteins. All GO terms shown are significantly enriched (FDR < 0.05). (**D**) Median abundances of the common cell cycle-regulated proteins across the cell cycle phases.

Fixed cells were sorted into G1, S, and G2&M populations, subjected to in-cell digestion (Kelly et al, 2022) and data-independent acquisition (DIA) LC-MS analysis (Fig. 2A and “Methods”). Cells were stained with an antibody against phosphorylated Rb S807/S811 (pRb) to identify early G1/G0 cells. However, nearly all mESC and NIH3T3 cells showed high pRb staining (data not shown), and therefore, we did not use pRb as a marker for FACS of these cell types. Because CD8 T cells had a significant fraction of quiescent cells with low pRb (as shown further below), CD8 T cells with high pRb staining were used to compare against the other cell lines.

In total, 8178 protein groups were identified with 2 or more peptides per protein. Replicates were highly correlated (Pearson’s *r* > 0.8), and correlation within a cell line was higher than between cell lines (Dataset EV1). Protein intensities were normalized by dividing the intensity of each protein by the summed total protein intensities per sample and multiplying by a million to generate normalized protein abundances in parts-per-million (ppm) units (Dataset EV2). We then compared these relative protein abundances between cell cycle phases and between cell types.

First, we systematically examined proteins that were cell-cycle-regulated. Using hierarchical clustering analysis, we identified a cluster of 121 proteins that were consistently cell cycle regulated across all three cell types (Dataset EV3; Fig. 2B). These proteins were highly enriched in annotated gene ontology (GO) terms associated with cell cycle functions (Fig. 2C), validating the PRIMMUS strategy. In general, the median protein abundance for these common cell cycle-regulated proteins in S and G2 phases were higher in the faster dividing CD8 T cells and mESCs (Fig. 2D). The median protein abundance in G1 phase for these proteins was higher in mESC compared to CD8 T cells and NIH3T3 (Fig. 2D). We concluded that many cell cycle-regulated proteins were periodic in mESC, and indeed, fluctuated to a similar degree compared to slower dividing NIH3T3 cells. However, significantly higher levels of these proteins were detected in G1 phase, consistent with previous reports of reduced APC/C activity in mESCs (Ballabeni et al, 2011).

### CD8 T cells and mESCs constitutively express Cyclin E1

**Figure 3:**
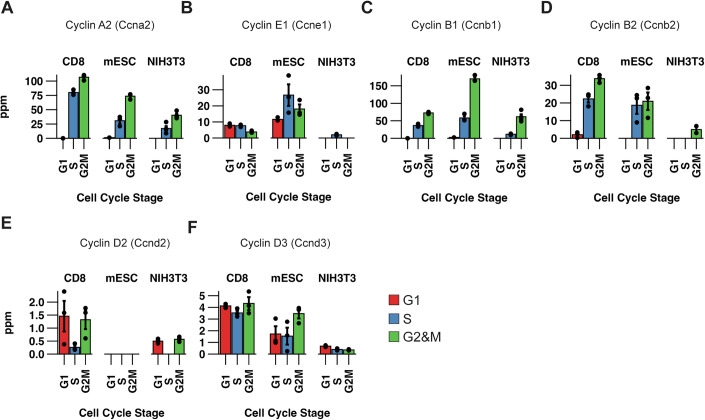
Comparing cell cycle regulation of Cyclin proteins in fast and slow dividing cell types. Protein intensities (ppm) for Cyclin A2 (**A**), Cyclin E1 (**B**), Cyclin B1 (**C**), Cyclin B2 (**D**), Cyclin D2 (**E**), and Cyclin D3 (**F**). Individual points are from distinct animals (CD8 T cells) or different passages (mESC, NIH3T3) (*n* = 3–4). The bar height indicates the mean, and error bars indicate the standard error of the mean (*n* = 3–4). For conditions in which a protein was not detected, no bar is shown.

Next, we examined specifically whether key proteins, including cyclins, were constitutively or periodically expressed in these cells. Cyclin A2 showed cell cycle-regulated abundance in all three cell populations (Fig. 3A). However, the abundances were generally higher in CD8 T cells and mESCs compared to NIH3T3. In NIH3T3 cells, Cyclin E1 levels were detected exclusively in S phase (Fig. 3B), consistent with Cyclin E1 degradation in S-phase (Clurman et al, 1996). In contrast, in mESCs and CD8 T cells, Cyclin E1 protein was robustly detected in G1, S, and G2&M phases, and at levels higher than maximally observed in NIH3T3 (Fig. 3B). Thus, while most proteins showed periodic fluctuation in all three cell lines, Cyclin E1 showed constitutive expression only in faster dividing cells suggesting Cyclin E1 as a candidate driver of rapid cell cycles.

Cyclin B1 and Cyclin B2 were cell cycle regulated in all three cell populations (Fig. 3C,D) and showed maximal abundance in G2&M. Cyclin B2 levels were lower in NIH3T3 cells compared to CD8 T cells and mESCs (Fig. 2D, ANOVA/Tukey’s *P* < 0.05). Cyclin B1 and Cyclin B2 show differential localization in HeLa cells, with Cyclin B2 localized to the Golgi apparatus and centrioles (Draviam et al, 2001; Spalluto et al, 2013). However, there is functional redundancy between the two B-type Cyclins (Hégarat et al, 2020). These results would be consistent with higher overall Cyclin B activity in faster dividing cells, or specialized functions of Cyclin B2, to promote CDK activity in Golgi and centrosomal compartments in CD8 T cells and mESC.

Cyclin D2 and Cyclin D3 showed cell-type-enhanced expression (Fig. 3E,F). Cyclin D2 was expressed in CD8 T cells and NIH3T3 (Fig. 3E). Cyclin D3 levels were higher in CD8 T cells and mESCs (Fig. 3F, *P* < 0.001). D-type cyclins in general are higher in abundance in CD8 T cells (Fig. 3E,F). The enhanced expression of Cyclin D3 in CD8 T cells is consistent with genetic studies showing tissue-specific defects in the development of the hematological system in the absence of all D-type cyclins and in T lymphocytes in Cyclin D3 knockout mice (Kozar et al, 2004; Sicinska et al, 2003).

**Figure EV1:**
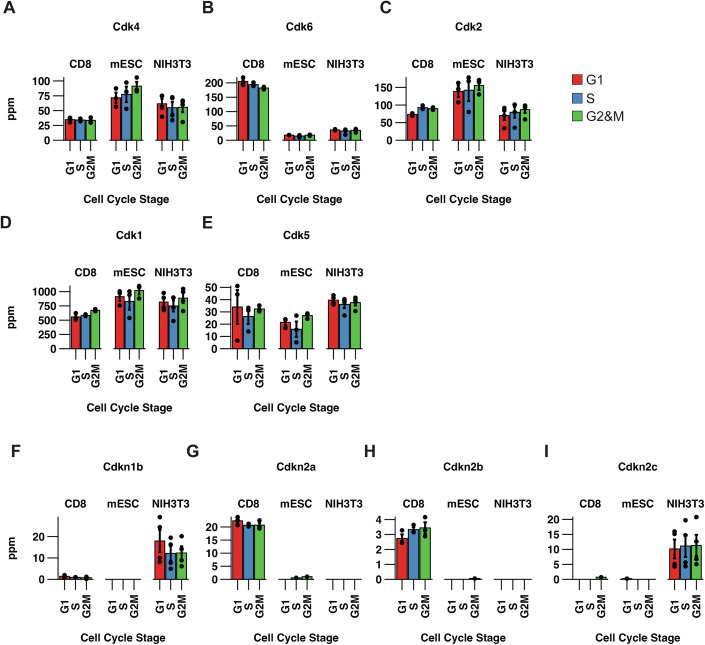
CDK and CDK inhibitor abundances. Protein intensities (ppm) for Cdk4 (**A**), Cdk6 (**B**), Cdk2 (**C**), Cdk1 (**D**), Cdk5 (**E**), Cdkn1b (p27) (**F**), Cdkn2a (p16) (**G**), Cdkn2b (p15) (**H**), and Cdkn2c (p18) (**I**). Individual points are from distinct animals (CD8 T cells) or different passages (mESC, NIH3T3). The bar height indicates the mean, and error bars indicate standard error of the mean (*n* = 3–4). For conditions in which a protein was not detected, no bar is shown.

Cyclins form active kinase complexes with cognate CDK proteins. CDK abundances typically do not show the same degree of periodic expression as Cyclin proteins. Consistent with this idea, our proteomics analysis showed relatively little to no variation in CDK protein abundance across the cell cycle (Fig. EV1A–E). However, CDKs showed cell-type-enhanced expression. For example, Cdk4 was higher expressed in mESC (Fig. EV1A, *P* < 0.05), whereas Cdk6 was higher expressed in CD8 T cells (Fig. EV1B, *P* < 0.001). These results suggest that the predominant complex between Cdk4/6 and D-type cyclins is Cdk6-Cyclin D3 in CD8 T cells and Cdk4-Cyclin D3 in mESC.

mESC also expressed higher levels of Cdk2 (Fig. EV1C, *P* < 0.001). Cdk1 levels were lower in CD8 T cells compared to the other two cell types (Fig. EV1D, *P* < 0.01). Interestingly, Cdk5 is expressed in CD8 T cells (Fig. EV1E) and was previously shown to be active in CD8 T cells and required for full TCR activation (Pareek et al, 2010). The cognate cyclin-like proteins for Cdk5 (p35/p39) were not detected in this dataset. However, recent results suggest that Cdk5 can form complexes with B-type cyclins (Zheng et al, 2024), which were expressed in CD8 T cells (Fig. 3C,D).

CDKs are inactivated by expression and binding to CDK inhibitor (CKI) proteins. We found cell-type enhanced expression of CKIs. For example, p27 (Cdkn1b) was detected in all cell cycle phases in NIH3T3 cells and CD8 T cells, albeit at much lower levels in CD8 T cells (Fig. EV1F). In contrast, p27 was not detectable in mESC. p16 (Cdkn2a) and p15 (Cdkn2b) are higher expressed in CD8 T cells (Fig. EV1G,H, *P* < 0.001 and *P* < 0.001, respectively), whereas p18 (Cdkn2c) was higher expressed in NIH3T3 (Fig. EV1I, *P* < 0.001). Comparing CKI levels to CDK and cyclin proteins, however, suggests that CKIs are likely to be present in relatively low, substoichiometric levels.

We conclude that the faster dividing CD8 T and mESC cell types are characterized by higher levels of canonical cell cycle-promoting cyclins, constitutive expression of Cyclin E1, and decreased levels of p27.

### CD8 T cells have high levels of the rate-limiting replication initiation factor Cdc45

At the start of the cell cycle, DNA replication origins must be loaded onto chromatin before S-phase entry. Origins are loaded in excess to enable firing of dormant origins following replication fork stalls to ensure timely S-phase completion. Previous work has suggested that G1 duration is dictated by the rate of DNA replication origin loading, and that increased abundance of DNA replication origin loading factors promotes rapid G1 progression in hESCs (Matson et al, 2017). Considering that mESCs and CD8s have a truncated G1 phase (Fig. 1), we next compared DNA replication origin licensing and initiation factors in our proteomics dataset.

**Figure 4:**
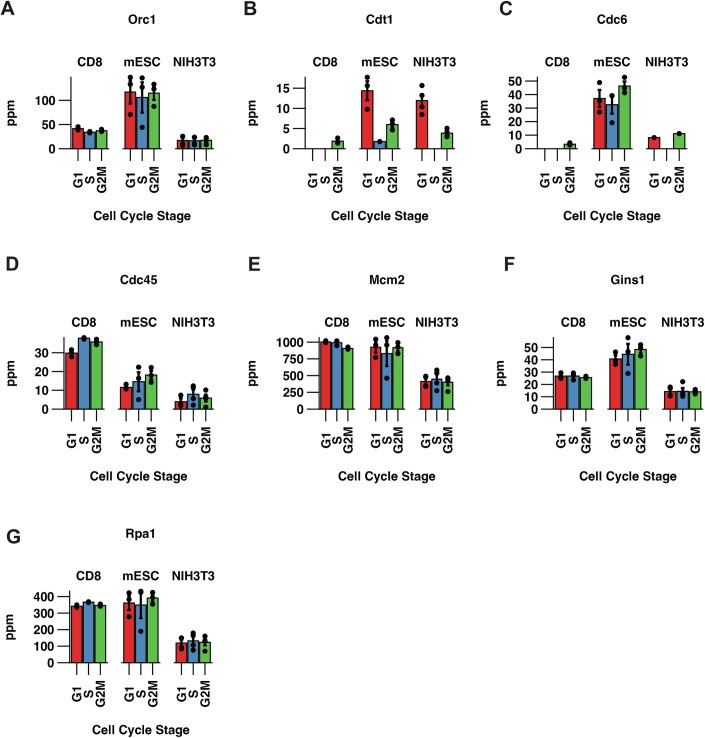
Comparing cell cycle regulation of Cdk and Cdk inhibitor proteins in fast and slow dividing cell types. Protein intensities (ppm) for Orc1 (**A**), Cdt1 (**B**), Cdc6 (**C**), Cdc45 (**D**), Mcm2 (**E**), Gins1 (**F**), and Rpa1 (**G**). Individual points are from distinct animals (CD8 T cells) or different passages (mESC, NIH3T3) (*n* = 3–4). The bar height indicates the mean, and error bars indicate standard error of the mean (*n* = 3–4). For conditions in which a protein was not detected, no bar is shown.

mESCs have higher levels of the Cdt1, Cdc6 and ORC (Orc1-6) proteins than NIH3T3 cells (Fig. 4A–C), consistent with previous work in hESCs. Only Orc1 is shown (Fig. 4A), but similar patterns are observed for Orc1-6 (Dataset EV2). Interestingly, CD8 T cells show decreased levels of Orc1 compared to mESCs and undetectable levels of Cdt1 and Cdc6. While not included in this analysis, Cdt1 and Cdc6 were detected in CD8 T cells with low pRb (data not shown), but at lower abundances compared to NIH3T3 cells. These results indicate that origin licensing factors are present in low abundance in CD8 T cells.

In contrast, in comparison to the other cell types, CD8 T cells have a higher abundance of proteins that form the Cdc45-MCM-GINS (CMG) helicase required for unwinding DNA for replication (Fig. 4D-F). For brevity, only Mcm2 and Gins1 are shown, but similar patterns are observed for the remaining subunits of the MCM2-7 and GINS1-4 heteromeric complexes (Dataset EV2). Of these factors, Cdc45 has been shown to be rate-limiting in yeast and mammalian cell lines (Wu and Nurse, 2009; Köhler et al, 2016; Tanaka et al, 2011). Interestingly, Cdc45 levels are highest in CD8 T cells, suggesting that more CMG helicases can be active simultaneously in this cell type, compared to NIH3T3. Ectopic overexpression of Cdc45 in HeLa cells results in more origin firing (Köhler et al, 2016). However, cells arrest in early S-phase arrest due to exhaustion of another crucial factor, the ssDNA-binding protein complex, RPA. RPA levels are more abundant in CD8 T cells compared to NIH3T3 (Fig. 4G). These results suggest that concomitant increases in Cdc45 and RPA may support increased origin firing in CD8 T cells without inducing replication catastrophe as observed in ectopic overexpression of Cdc45 alone.

### Cyclin E1 regulation in CD8 T cells

**Figure 5:**
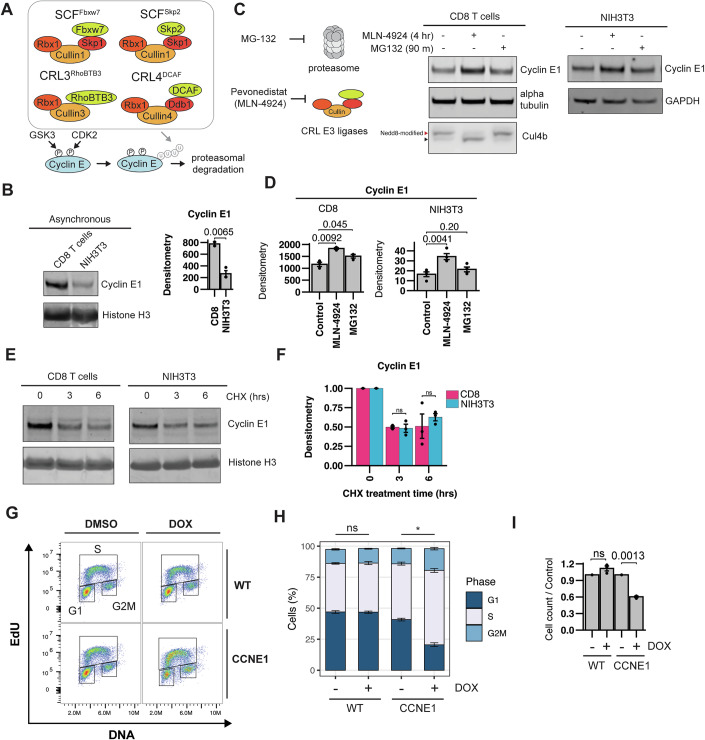
Regulation of Cyclin E1 abundance in CD8 T cells. (**A**) Pathways that target Cyclin E for degradation. (**B**) Cell lysates from asynchronous activated OT-1 CD8 T cells and NIH3T3 cells were assayed by immunoblot for Cyclin E1. Densitometry shown is normalized for loading using Histone H3. The *P* values shown are from pairwise *t* tests (*n* = 3 biological replicates), error bars indicate s.e.m. (**C**) Activated CD8 T cells and NIH3T3 cells were treated with vehicle, 25 µM MG-132 for 90 min and 1 µM MLN-4924 for 4 h and assayed for Cyclin E1 protein levels by immunoblot. (**D**) Densitometry of the immunoblot shown in (**C**). Intensities are normalized for loading using alpha tubulin and GAPDH. Individual points are from distinct animals (CD8 T cells) or different passages (NIH3T3). The bar height indicates the mean, and error bars indicate the standard error of the mean (*n* = 3). *P* values are from pairwise *t* tests. (**E**) Activated CD8 T cells and NIH3T3 cells were treated with cycloheximide (CHX) for the indicated times and immunoblotted for Cyclin E1. (**F**) Densitometry of immunoblot shown in (**E**). Intensities are normalized for loading using Histone H3. A repeated-measures ANOVA was used to calculate statistical significance. (*n* = 3 biological replicates, error bars indicate s.e.m.) (**G**) Cell cycle frequency analysis of parental NIH3T3 (WT) and NIH3T3 CCNE1^OE^ cells treated with DMSO or 20 ng/ml doxycycline (DOX) for 3 days using flow cytometry. (**H**) Quantitation of (**G**). Error bars indicate the standard error of the mean. The asterisk (*) indicates that all *P* values were less than 0.05 for all phases comparing DMSO versus DOX (*t* test). ns indicates not significant (*P* > 0.05), *n* = 3 biological replicates. (**I**) Relative cell counts from experiment shown in (**G**) normalized to -DOX control. The *P* value shown is from a pairwise *t* test, *n* = 3 biological replicates, error bars indicate s.e.m. Source data are available online for this figure.

Cyclin E1 overexpression has been shown to induce rapid G1 progression and an increase in proliferation in multiple mammalian cell lines. Constitutive, high expression of Cyclin E1 in CD8 T cells and mESCs could be key in promoting short G1 phases in these two cell types. An important question is therefore: why is there high Cyclin E1 protein in CD8 T cells? In this context, Cyclin E contains a phosphodegron (Clurman et al, 1996; Welcker et al, 2003) sequence that is recognized by the cullin ring ligase (CRL) SCF^Fbxw7^, resulting in ubiquitination and proteasome-mediated degradation. Cyclin E1-CDK2 itself phosphorylates Cyclin E1 to produce the Cyclin E phosphodegron. Thus, high Cyclin E1-CDK2 activity results in increased Cyclin E1 degradation. Importantly, this negative feedback loop contributes to cell cycle-regulated fluctuation in Cyclin E1 abundance. SCF (Skp2) (Nakayama et al, 2000) and CRL3 (RhoBTB3) (Lu and Pfeffer, 2013) are two additional cullin-ring ligase (CRL) complexes that putatively target Cyclin E for degradation. More recently, Cyclin E2, but not Cyclin E1, was expressed at higher levels in Cul4b knockout T cells (Dar et al, 2023), consistent with CRL4-mediated degradation (Higa et al, 2006). These pathways are summarized in Fig. 5A.

**Figure EV2:**
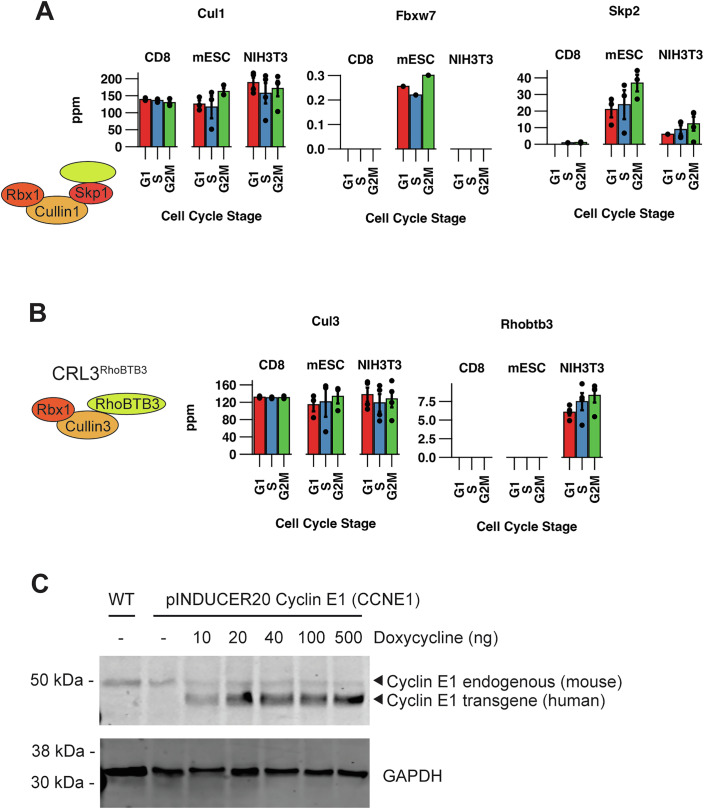
Cyclin E1 regulation and overexpression. Protein intensities (ppm) for Cul1, Fbxw7, and Skp2 (**A**), and Cul3 and Rhobtb3 (**B**). Individual points are from distinct animals (CD8 T cells) or different passages (mESC, NIH3T3). The bar height indicates the mean, and error bars indicate standard error of the mean (*n* = 3–4). For conditions in which a protein was not detected, no bar is shown. (**C**) Immunoblot showing expression of Cyclin E1 transgene following doxycycline treatment of NIH3T3 CCNE1^OE^ cells. Source data are available online for this figure.

We first orthogonally validated that Cyclin E1 levels were higher in CD8 T cells compared to NIH3T3 by immunoblot analysis of protein lysates from asynchronous cultures (Fig. 5B). To identify mechanisms leading to higher Cyclin E1 levels in CD8 T cells, we examined CRL levels in the proteomic dataset. While Cul1 and Cul3 levels are similar across the three cell types (Fig. EV2A,B), Fbxw7 and Skp2 are very low or undetectable in CD8 T cells and NIH3T3 cells (Fig. EV2A). RhoBTB3 is highly expressed in NIH3T3 cells and undetectable in CD8 T cells (Fig. EV2B). These data suggest CD8 T cells have reduced levels of known CRL substrate adapter proteins for Cyclin E1.

We next tested if Cyclin E1 regulation by CRL- and proteasome-dependent degradation differs between CD8 T cells and NIH3T3 cells. 90-min treatment with the proteasome inhibitor MG-132 significantly increased Cyclin E1 levels (Fig. 5C,D), suggesting that Cyclin E1 protein is subject to proteasome-mediated degradation. We next tested if CRL activity is required for Cyclin E1 degradation. Four-hour treatment with the small molecule MLN-4924, which blocks CRL activation, significantly increased Cyclin E1 levels (Fig. 5C,D). Treatment of NIH3T3 cells with MG-132 and MLN-4924 also resulted in stabilization of Cyclin E1, to a similar extent to CD8 T cells (Fig. 5C,D). We conclude that Cyclin E1 is degraded in CD8 T cells and NIH3T3 cells in a proteasome- and Neddylation-dependent manner, likely via a CRL.

We next compared Cyclin E1 protein stability in CD8 T and NIH3T3 cells. Treatment with cycloheximide (CHX), which blocks protein synthesis, resulted in decreased Cyclin E1 levels over time, consistent with a ~3-h protein half-life (Fig. 5E,F). The half-life of Cyclin E1 was not significantly different between CD8 T cells and NIH3T3 cells (Fig. 5F). We conclude that the increased Cyclin E1 observed in CD8 T cells is unlikely to be due to major differences in protein stability. Instead, these results suggest the differences are more likely due to differences in transcriptional or translational regulation of Cyclin E1 expression. Consistent with this idea, the average Cyclin E1 mRNA abundance (in transcripts per million, or TPM) is approximately tenfold higher in lymphoid cell lines compared to fibroblast cell lines (Broad, 2025).

### Cyclin E1 overexpression in NIH3T3 increases S-phase frequencies and reduces cell numbers

To investigate if the difference in Cyclin E1 levels between these three cell types is causal for differences in cell cycle speed, we tested if Cyclin E1 overexpression could enhance NIH3T3 proliferation. We generated a NIH3T3 stable cell line containing a doxycycline-inducible Cyclin E1 transgene (NIH3T3 CCNE1^OE^). Transgene expression following doxycycline (DOX) treatment was confirmed by immunoblot (Fig. EV2C). At 20 ng DOX, the ratio of exogenous to endogenous Cyclin E1 in NIH3T3 CCNE1^OE^ cells (Fig. EV2C) was similar to that ratio between CD8 T and NIH3T3 cells (Fig. 5B). Therefore, 20 ng DOX was used for the remaining experiments. Parental NIH3T3 (WT) and NIH3T3 CCNE1^OE^ cells were treated with DOX for 72 h and assayed for cell cycle frequencies by flow cytometry (Fig. 5G). DOX treatment caused a decrease in G1 frequency, and an increase in S- and G2&M-phase frequencies (Fig. 5H) in CCNE1^OE^ but not WT cells, consistent with high Cyclin E1 levels promoting rapid G1 progression. Interestingly, this change in cell cycle phase frequency did not result in increased cell numbers. Instead, Cyclin E1 overexpression reduced cell numbers (Fig. 5I). This may be due to premature S-phase entry with reduced replication origins and replication stress in S-phase, as shown previously (Matson et al, 2017). Consistent with replication stress-induced arrest, there is an increase in G2&M frequencies (Fig. 5I). These data suggest that while high levels of Cyclin E1 can promote rapid S-phase entry, additional mechanisms are required for CD8 T cells to avoid replication stress-induced arrest.

### APC/C regulation in CD8 T cells

Numerous cell cycle-regulated proteins are targeted for degradation in late mitosis and G1 phase by the APC/C E3 ligase (Pines, 2011; Buschhorn and Peters, 2006; Primorac and Musacchio, 2013), including Cyclin A2 and Cyclin B1. It has been previously suggested that cell type-specific regulation of the APC/C E3 ligase underpins subdued periodicity in cyclin proteins in embryonic stem cells (Ballabeni et al, 2011). We next investigated if APC/C substrates, subunits, and regulators showed cell-type-specific expression.

To examine a proposed model whereby APC/C activity is reduced in the G1 phase in mESC (Ballabeni et al, 2011; Padgett and Santos, 2020), we analyzed APC/C substrates (Davey and Morgan, 2016) detected in this study (Dataset EV4). 15 substrates do not show cell cycle-regulated behavior in any of the three cell types studied. For example, GLS was characterized as an APC/C substrate in neurons (Herrero-Mendez et al, 2009). However, GLS protein was not increased in G2&M compared to G1 in NIH3T3, as would be expected for a bona fide APC/C substrate (Dataset EV2). These 15 proteins were excluded from the analysis.

**Figure 6:**
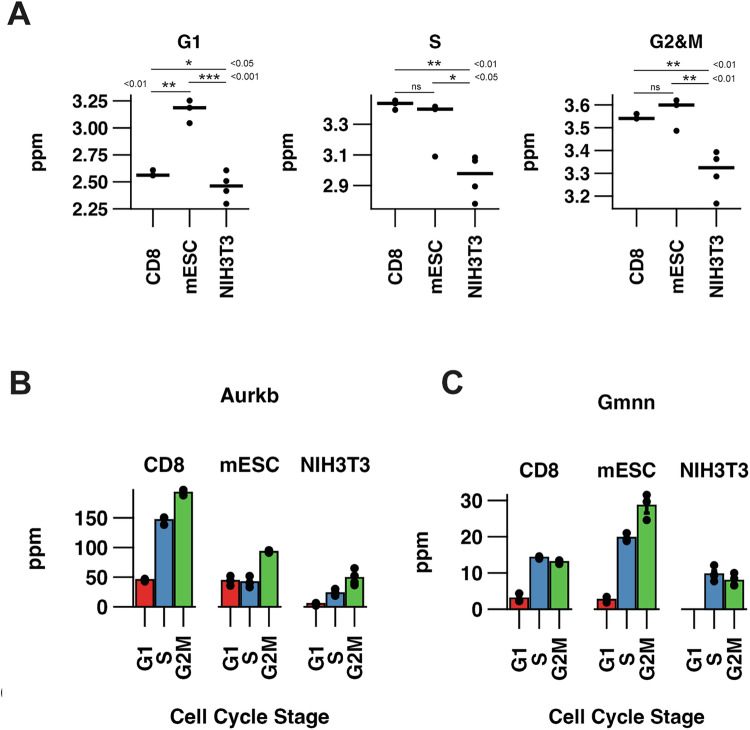
Variation in APC/C substrate abundance across cell types. (**A**) Summed protein intensities for APC substrates (Davey and Morgan, 2016) detected by proteomics (Dataset EV4). Individual points are from distinct animals (CD8 T cells) or different passages (mESC, NIH3T3) (*n* = 3–4). Lines indicate the mean. Statistical significance was tested using ANOVA and Tukey’s test. Significance is denoted by *P < 0.05, **P < 0.01, and ***P < 0.001. Protein intensities (ppm) for Aurkb (**B**) and Gmnn (**C**). Individual points are from distinct animals (CD8 T cells) or different passages (mESC, NIH3T3). The bar height indicates the mean, and error bars indicate the standard error of the mean (*n* = 3–4). For conditions in which a protein was not detected, no bar is shown.

We then assessed the abundance of the remaining 43 APC/C substrates between cell lines (Fig. 6A). To do this, we summed substrate abundances as a readout of total APC/C substrate levels. In G1, the summed APC/C substrate abundance was significantly higher in mESC and CD8 T cells than in NIH3T3, and mESC showed the highest abundance overall. For example, Aurora kinase B (Aurkb) and Geminin (Gmnn) showed higher levels in mESC and CD8 T cells in G1 (Fig. 6B,C, red bars). In S and G2 phases, APC/C substrates were present at higher levels in CD8 T cells and mESC. Thus, our data suggest APC/C activity is reduced in mESCs and CD8 T cells.

### Emi1 promotes G1/S progression in CD8 T cells

**Figure 7:**
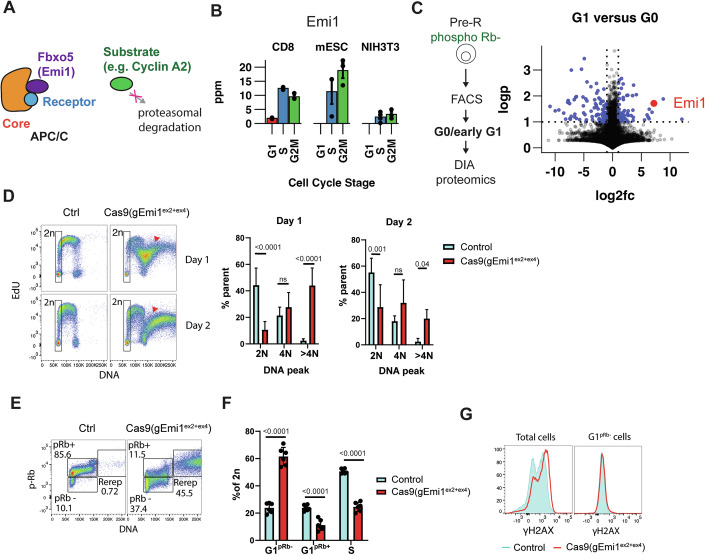
Emi1 promotes S-phase entry and safeguards against re-replication. (**A**) Scheme illustrating Emi1/Fbxo5 molecular function. (**B**) Protein intensities (ppm) for Emi1 (*n* = 3–4 biological replicates). For conditions in which a protein was not detected, no bar is shown, error bars indicate s.e.m. (**C**) G0/early G1 CD8 T cells were isolated by FACS and analyzed by quantitative proteomics. Volcano plot showing log_0.05_ *P* values (*t* test) and log_2_ fold changes. Significantly changing proteins are highlighted in purple, and Emi1 is shown in red. (**D**) CD8 T cells were activated and cultured for 2 days prior to electroporation with Cas9-gRNA ribonucleoprotein complexes targeting Emi1. Two gRNAs targeting exons 2 and 4 were pooled. Following treatment with Cas9(gEmi1^ex2+ex4^), cells were cultured for 1 or 2 more days for analysis. Prior to harvest, cells were given a 30 min pulse of EdU. Cells were analyzed by flow cytometry. Frequency of cells containing >4N DNA. Red arrows indicate re-replicating cells. (*n* = 6, representing distinct animals, collected over two time periods, error bars indicate s.e.m.), (**E**) Representative flow cytometry staining of phosphorylated Rb (phospho-S807/811) comparing control cells and cells treated with Cas9(gEmi1^ex2+ex4^). (**F**) Frequency of early G1/G0 (G1^pRb-^), late G1 (G1^pRb+^), and early S-phase cells at day 2 following Cas9 treatment. Only cells within the 2N DNA content gate as indicated in (**B**) are shown. (*n* = 6, representing distinct animals, collected over two time periods, error bars indicate s.e.m.), (**G**) γH2AX expression was assessed either in all cells or G1^pRb-^ cells at day 2 following Cas9 treatment. DNA staining was determined by Hoechst. Statistical significance was determined by unpaired *t* tests, *n* = 6 mice from two independent cohorts. Source data are available online for this figure.

Emi1 facilitates rapid mESC cell divisions by inhibiting the APC/C and stabilizing cell cycle promoting proteins, including Cyclin A2 (Ballabeni et al, 2011) (Fig. 7A). While we did not observe increased Cyclin A2 levels in G1 phase in mESCs (Fig. 3A), the levels of other APC/C substrates were higher in G1 phase in mESC and CD8 T cells (Fig. 6A–C). Emi1 protein levels were highest in CD8 T cells and mESCs (Fig. 7B). Notably, Emi1 was not detected in published proteomic analyses of human NB4 promyelocytes (Ly et al, 2015, 2014, 2017), human TK6 lymphoblasts (Kelly et al, 2022) and human HeLa S3 epithelial cells (Aviner et al, 2015). To test if Emi1 levels are regulated during G1, we performed a proteomic analysis comparing G0/early G1 cells and late G1 cells using pRb as a marker. Emi1 was a top hit showing higher levels in late G1 cells (Fig. 7C). We next took advantage of a characteristic of ex vivo CD8 T cells, whereby activated cells slow proliferation following prolonged treatment with the IL-15 cytokine as compared with IL-2. Consistent with a role of Emi1 in proliferation timing, the ImmPRes database showed that IL-15-treated cells had decreased Emi1 levels compared to IL-2-treated cells (Brenes et al, 2023). Moreover, there is significant overlap between APC/C substrates and E2F targets, including Emi1 itself, thereby making it challenging to ascertain whether high Emi1 levels cause or is a consequence of rapid cell cycles. It is therefore important to test if Emi1 and reduced APC/C activity play a causal role.

**Figure EV3:**
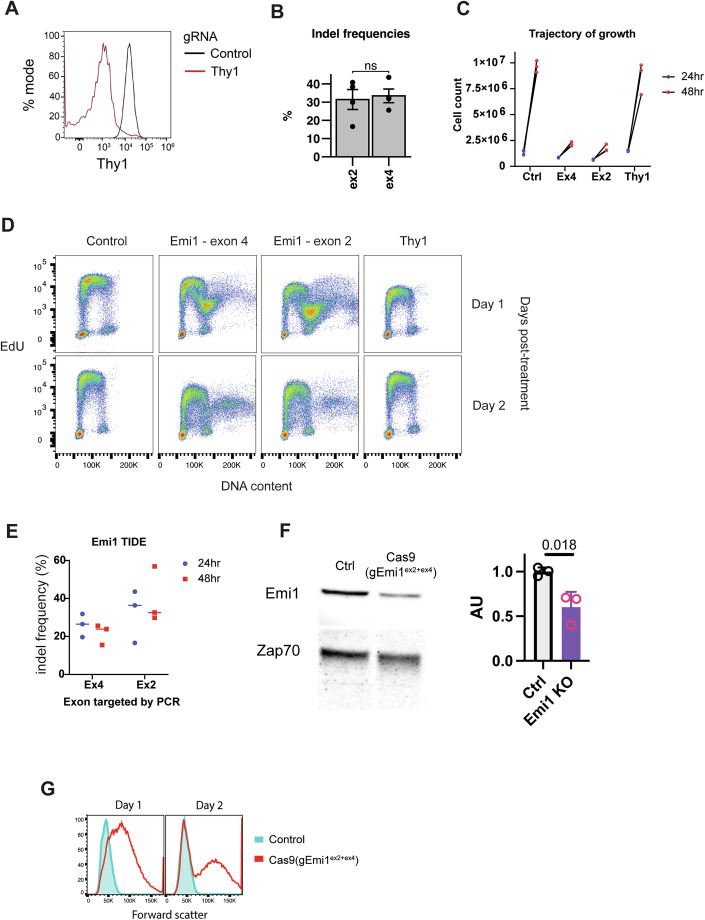
Emi1 promotes S-phase entry and safeguards against re-replication. (**A**) CD8 T cells treated with Cas9(gThy1^ex3^), stained with anti-Thy1 antibody, and analyzed by flow cytometry. (**B**) CD8 T cells treated with either Cas9(gEmi1^ex2^) or Cas9(gEmi1^ex4^) and cultured for 24 h were harvested for genomic DNA. Two gRNAs were tested, which target exon 4 (Ex4) and exon 2 (Ex2) of the Emi1/Fbxo5 locus. Indel frequencies were measured by PCR and TIDE analysis. (*n* = 3 individual animals), statistical significance assessed using *t* test. (**C**) Cell counts following control (electroporation with no Cas9), Cas9(gEmi1^ex2^), Cas9(gEmi1^ex4^), or Cas9(gThy1^ex3^) treatment and culturing cells for 24 and 48 h. (*n* = 3 individual animals) (**D**) Cell cycle analysis of cells as in (**C**). (**E**) TIDE analysis of cells treated with Cas9(gEmi1^ex2+ex4^). (*n* = 3 individual animals), (**F**) Immunoblot analysis of extracts from control and Cas9(gEmi1^ex2+ex4^) treated cells, probing for Emi1 protein. Zap70 was probed to assess loading. (*n* = 3 individual animals), statistical significance assessed using *t* test. (**G**) Forward scatter measurements of control and Cas9(gEmi1^ex2+ex4^) treated cells. Forward scatter is indicative of cell size. Source data are available online for this figure.

To do this, we deleted Emi1 in CD8 T cells by electroporating preformed Cas9-guide RNA (gRNA) ribonucleoprotein (RNP) complexes. To test the approach efficiency, we introduced Cas9-gRNAs targeting exon 3 of the fitness-neutral cell surface receptor Thy1, i.e., Cas9(gThy1^ex3^), into CD8 T cells. This resulted in an efficient decrease in Thy1 expression (Fig. EV3A). Whole cell population deletion of essential genes, like Emi1, can be challenging for fast-dividing CD8 T cells because minor cell subpopulations containing the intact locus can rapidly outgrow other cells in the population in which gene disruption has occurred. Despite these limitations, we tested this approach to perturb Emi1 function in primary ex vivo CD8 T cells. Two Cas9-gRNA RNPs were used, containing gRNAs that target exons 2 and 4 of Emi1, abbreviated as Cas9(gEmi1^ex2^) and Cas9(gEmi^ex4^). Introduction of either Cas9(gEmi1^ex2^) or Cas9(gEmi^ex4^) in activated CD8 T cells resulted in indels in the Emi1 locus (Fig. EV3B) and reduced proliferation (Fig. EV3C). These cells underwent re-replication as indicated by the presence of cells with >4 N DNA content (Fig. EV3D), a phenotype observed previously in mammalian cell lines (Machida and Dutta, 2007; Di Fiore and Pines, 2007). We next tested introducing a combination of both RNPs to increase gene disruption efficiency. Cells treated with Cas9(gEmi1^ex2^)+Cas9(gEmi^ex4^), abbreviated as Cas9(gEmi1^ex2+ex4^), resulted in a stronger re-replication phenotype (Fig. 7D). Therefore Cas9(gEmi1^ex2+ex4^) was used for all subsequent experiments. Treatment with Cas9(gEmi1^ex2+ex4^) resulted in indels in the Emi1 locus and reduced Emi1 protein levels as shown by immunoblotting (Fig. EV3E,F). Cells treated with Cas9(gEmi1^ex2+ex4^) were also larger in cell size compared to control cells, as inferred from forward scatter measurements (Fig. EV3G). These results confirm that Cas9(gEmi1) RNPs perturb Emi1 function in CD8 T cells.

While the major effect of Emi1 deletion is re-replication (Fig. 7D), to investigate the role of Emi1 specifically in G1/S, we excluded cells with >4N DNA and co-stained for pRb to identify pre- and post-restriction point G1 cells (G1^pRb-^ and G1^pRb+^). In cells that have not undergone DNA re-replication, the frequency of pRb^−^ cells increased from 10% in control cells to 37% in Cas9(gEmi1^ex2+ex4^) treated cells (Fig. 7E). Indeed, in cells with 2N DNA content (gate shown in Fig. 7D), there was an increase from 23% G1^pRb-^ in control cells to 60% G1^pRb-^ in Cas9(gEmi1^ex2+ex4^) treated cells, and corresponding decreases in G1^pRb+^ and early S-phase (EdU + ) (Fig. 7F). To examine the possibility that increased frequency of G1^pRb-^ is due an abortive S-phase and return to G1 following DNA damage, we stained for the DNA damage marker gammaH2A.x. In cells ungated for cell cycle phase, we observed an increase in γH2A.x staining, consistent with DNA damage induced by Emi1 gene disruption in S-phase cells (Fig. 7G, left). However, G1^pRb-^ cells showed no difference in γH2A.x intensity between control and Cas9(gEmi1^ex2+ex4^) treated cells (Fig. 7G, right). This suggests that the increased frequency of G1^pRb-^ cells and decreased S-phase frequency observed in Cas9(gEmi1^ex2+ex4^) treated cells is likely due to a function of Emi1 in promoting G1 progression and S-phase entry.

Together, these observations support the model that, like in mESCs, Emi1 facilitates cell cycle progression in CD8 T cells.

### Cell cycle-promoting factors are similarly expressed in cycling memory-like and effector CD8 T cells

**Figure 8:**
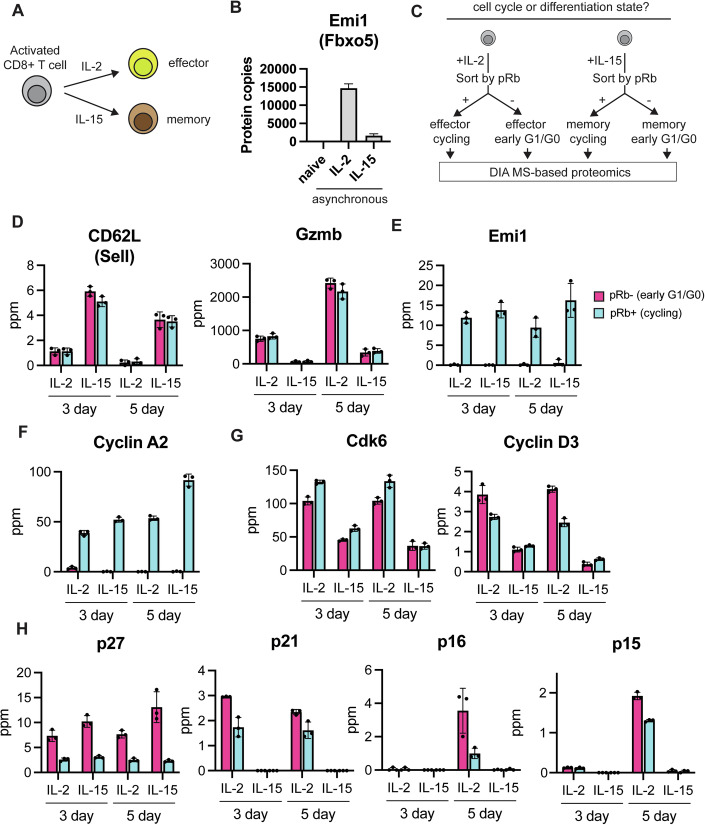
Emi1 is cell cycle regulated and promotes S-phase entry in both CD8 T effector and memory-like cells in vitro. (**A**) Schematic showing CD8 T cell phenotype specification in vitro by the IL-2 and IL-15 cytokines. (**B**) Emi1/Fbxo5 protein copies measured in activated CD8 T cells cultured in IL-2 versus IL-15 (*n* = 3 biological replicates, error bars indicate s.e.m.) (Brenes et al, 2023). (**C**) Schematic illustrating the approach to compare cell cycle-regulated protein networks in effector and memory-like CD8 T cells. OT-1 CD8 T cells were activated with antigen (N4 peptide) for 2 days, and then cultured for an additional 2 days with indicated cytokine (IL-2 or IL-15) prior to harvest and analysis by data-independent acquisition (DIA) MS-based quantitative proteomics. For all subsequent plots, error bars indicate s.e.m. (**D**) Protein abundance (ppm) of selected CD8 T cell phenotypic markers CD62L (Sell) and granzyme B (Gzmb) (*n* = 3 biological replicates). (**E**) Protein abundance (ppm) of Emi1/Fbxo5 (*n* = 3). (**F**) Protein abundance (ppm) of Cyclin A2 (*n* = 3 biological replicates). (**G**) Protein abundance (ppm) of positive regulators of G1/S transition, Cdk6 and Cyclin D3 (*n* = 3 biological replicates). (**H**) Protein abundance (ppm) of negative regulators of cell cycle progression, including members of the CIP/KIP family (p21, p27) and INK4 family (p16, p15), *n* = 3 biological replicates. For conditions in which a protein was not detected, no bar is shown.

**Figure EV4:**
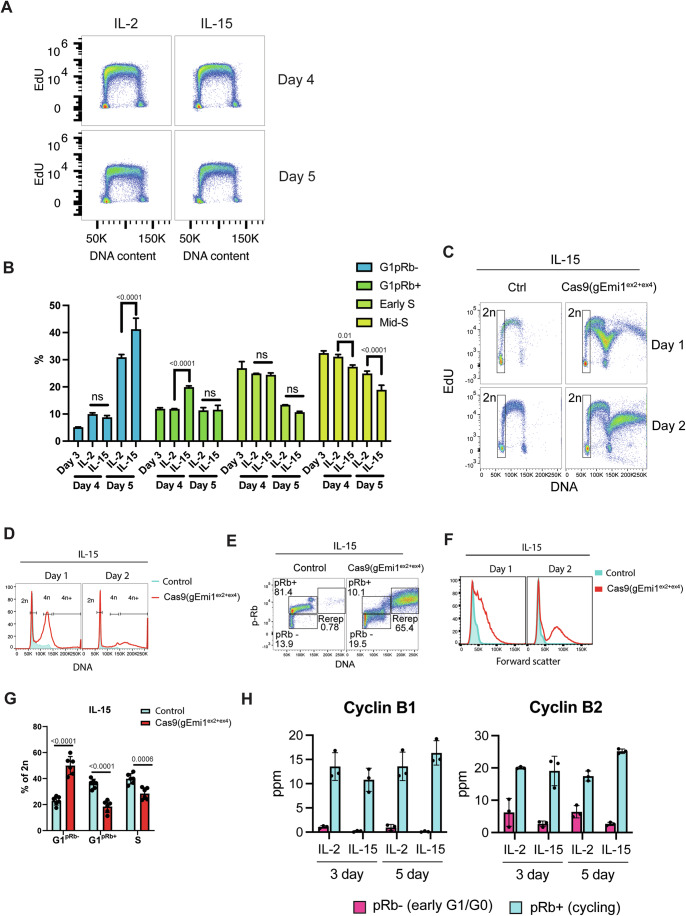
Role of Emi1 in memory-like CD8 T cells. (**A**) CD8 T cells were activated for two days and then cultured either in IL-2 or IL-15 for an additional 2 or 3 days. Cell cycle distribution was measured using phosphorylated Rb, EdU, and DNA content. Representative cell cycle profiles are shown. (**B**) Calculated frequencies from data shown in (**A**). Total time post activation is shown on the *x* axis. (**C**) EdU incorporation of CD8 T cells activated at day 0, treated with Cas9(gEmi1^ex2+ex4^) or a blank control on day 2, cultured IL-15, and cultured for an additional one or two days prior to harvest for analysis. (**D**) DNA content histograms of cells from (**C**); (**E**) Rb phosphorylation of cells from (**C**). (**F**) Forward scatter measurements by flow cytometry of cells from (**C**); (**G**) quantitation of early G1/G0 (G1^pRb-^), late G1 (G1^pRb+^), and early S-phase cells of cells from (**C**). Statistical significance was determined by unpaired *t* tests, *n* = 6 mice from two independent cohorts (**A**–**F**), *n* = 3 mice (**G**). (**H**) Protein abundance (ppm) of Cyclin B1 and Cyclin B2 comparing IL-2 and IL-15 treated cells (*n* = 3). Source data are available online for this figure.

CD8 T cells are multipotent (Kaech et al, 2002). Following activation, CD8 T cells can differentiate into effector and memory phenotypes that have functions in pathogen elimination and long-term immunity, respectively. Differentiation can be promoted in vitro by culturing activated CD8 T cells with cytokines: IL-2 for effector cells and IL-15 for “memory-like” cells (Fig. 8A). Cells treated for 1 day with IL-2 or IL-15 show similar cell cycle profiles (Fig. EV4A). However, by 3 days, cytokine treatment has a significant impact on cell cycle distribution (Fig. EV4B). Compared to IL-2-treated cells, IL-15-treated cells have a higher frequency of G1^pRb-^ cells (Fig. EV4B).

Interestingly, levels of Emi1 were significantly lower in asynchronous IL-15-treated cells compared to asynchronous IL-2 cells (Fig. 8B) (Brenes et al, 2023). Because Emi1 facilitated G1 progression (Fig. 7), it is possible that lower Emi1 levels resulted in slower cell division cycles in IL-15-treated cells. Alternatively, it is equally possible that because Emi1 levels are cell cycle regulated (Fig. 7B), the difference in Emi1 levels could be explained by differences in cell cycle phase frequency following prolonged culture (Fig. EV4B).

To distinguish between these possibilities, IL-2 and IL-15-treated cells were FACS separated into pRb^−^ and pRb^+^ populations, which correspond to early G1/G0 and cycling cells, respectively, and total protein levels were measured proteome-wide (Fig. 8C). CD62L, also known as L-selectin (encoded by the *Sell* gene), and additional markers (e.g., Ccr7, Tcf1, Eomes, etc.), are frequently used to identify memory phenotype. CD62L was highest in IL-15-treated cells (Fig. 8D). Granzyme B (Gzmb) is a marker of effector phenotype and was highest in IL-2-treated cells (Fig. 8D). These results are therefore consistent with IL-2 and IL-15 driving effector and memory-like differentiation, respectively. Emi1 levels, however, were consistently low in pRb^−^ cells and high in pRb^+^ cells, irrespective of cytokine treatment (Fig. 8E). This suggests the difference in Emi1 levels observed in asynchronous IL-15 versus IL-2 treated cells (Fig. 8B) likely reflected a change in the cell cycle phase distribution promoted by cytokine treatment.

These observations raised the question of whether Emi1 functions similarly in IL-15-treated cells to promote G1 progression. Cas9(gEmi1^ex2+ex4^) treatment in IL-15 cells induced re-replication (Fig. EV3C–E), an increase in cell size (Fig. EV3F), and increased G1^pRb-^ cell frequency (Fig. EV3G), resembling the phenotype of Cas9(gEmi1^ex2+ex4^) treated IL-2 cells shown in Fig. 7. This similarity suggests that Emi1 acts downstream of the critical factors that control cell cycle entry following differential cytokine treatment and instead promotes rapid S-phase entry post-commitment.

Interestingly, like Emi1, A- and B-type cyclins fluctuated by similar or even higher fold changes in IL-15 versus IL-2 treated cells (Figs. 8F and EV3H). These data suggest that once cells pass the restriction point and become committed to cell cycle progression, IL-15-treated cells spend a similar time to IL-2-treated cells completing a cell division cycle. These results are consistent with early reports showing that IL-15 and IL-2 can induce similar levels of proliferation (Cornish et al, 2006).

Cdk6 and Cyclin D3 were notable exceptions to the general trend exemplified by Cyclin A2. Both Cdk6 and Cyclin D3 were higher in IL-2-treated cells compared to IL-15-treated cells (Fig. 8G). Examination of CDK inhibitor proteins of the CIP/KIP (CDK interacting protein/kinase inhibitory protein) and INK4 (inhibitor of CDK4) family showed differential expression patterns. The CIP/KIP protein p27 was higher in pRb^-^ compared to pRb^+^ cells, irrespective of cytokine treatment. This lack of cytokine-dependence for p27 resembles other core cell cycle proteins, like Emi1 and Cyclin A2. However, expression of p21, p16, and p15 was specific to IL-2-treated cells. p16 and p15 are associated with terminal cell cycle exit and cellular senescence (Herranz and Gil, 2018). Interestingly, p16 and p15 increased in expression with time in culture with IL-2 (Fig. 8H), suggesting that CD8 T cells may acquire senescent-like characteristics following prolonged culture in IL-2 but not IL-15.

## Discussion

The structure and control of the cell division cycle are developmentally plastic to facilitate the differentiation of embryonic stem cells into somatic cell types that vary in propensity for proliferation. Unlike many other cells in an adult mammal (Tomasetti and Vogelstein, 2015), cells of the adaptive immune system (B and T cells) retain high proliferation potential and repeatedly undergo several rounds of rapid proliferation followed by dormancy, for example, during somatic maturation, acute viral responses, and anti-cancer immunity.

The mechanisms adaptive immune cells employ to promote rapid proliferation remain poorly understood. To investigate these mechanisms, we have performed a quantitative comparison of cell cycle-regulated protein abundance in three conspecific cell types (CD8 T cells, mESCs, and NIH3T3 fibroblasts) that vary in cell cycle speed. We have used PRIMMUS (Ly et al, 2017; Kelly et al, 2022), an approach that avoids arrest-based synchronization methods, which can promote differentiation of multipotent cells and arrest-induced stress responses that are not related to cell cycle per se.

### Periodic expression of Cyclin A2 is a common feature of cycling cells

Constitutive expression of specific cell cycle regulators has been proposed as a mechanism to promote rapid proliferation in early embryonic cell cycles (Padgett and Santos, 2020). In contrast to previous reports that Cyclin A2 is either constitutively expressed or has dampened periodicity in mESCs, our data show that Cyclin A2 levels are highly periodic in the three cell lines examined. Our results suggest that differences in Cyclin A2 regulation are unlikely to explain variation in cell cycle duration.

### Multiple shared adaptations of embryonic and CD8 T cell cycles

Our results instead suggest that rapid cell divisions may be promoted by multiple mechanisms, including constitutive expression of Cyclin E1, reduced levels of CDK inhibitor proteins, and quantitatively higher levels of A-, B-, and D-type Cyclin proteins. Faster dividing cells also express higher levels of the Usp37 deubiquitinase (Dataset EV2) and Emi1, both proteins that antagonize or directly inhibit APC/C-mediated degradation of cell cycle-promoting proteins. Our results suggest that multiple adaptations to cell cycle control likely contribute to CD8 T-cell proliferation, consistent with selective pressure for rapid immune cell expansion.

### Distinctive features of CD8 T-cell cycles

Although mESCs and CD8 T cells share many similarities in cell cycle structure and mechanistic adaptations, we also find striking differences. For example, CD8 T cells have a significantly lower frequency of G2&M cells, suggesting that G2 phase duration is shorter. Previous work has shown that CD8 T cell proliferation is unaffected by p21 overexpression, for reasons that remain unclear. Considering p21 is important in eliciting G2 arrest, it will be important to test if p21 insensitivity contributes to reduced G2 duration in CD8 T cells.

Rapid proliferation of hESCs was shown to be dependent on fast replication origin licensing during G1 due to high abundance of the Cdt1 loading factor (Matson et al, 2017). While high Cdt1 abundance was observed in mESCs, Cdt1 levels were below detectable limits in CD8 T cells in G1 phase. CD8 T cells similarly showed reduced levels of the Cdc6 loading factor. Instead, CD8 T cells showed increased levels of Cdc45, the rate-limiting factor in DNA replication origin firing. It will be interesting in the future to directly measure DNA replication origin loading and firing in CD8 T cells.

### Cyclin E1 shows constitutive expression in rapidly cycling cells

Of the cell cycle cyclins and CDKs examined, only Cyclin E1 showed constitutive expression across the cell cycle in mESCs and CD8 T cells. This is consistent with earlier work showing that Cyclin E-Cdk2 activity is consistently high in ESC and becomes periodic following loss of pluripotency. Considering that overexpression of Cyclin E1 is sufficient to reduce cell cycle duration (Ohtsubo and Roberts, 1993), our results suggest that CD8 T cells also utilize Cyclin E1 to drive rapid immune cell clonal expansion. Because Cyclin E1 overexpression causes chromosomal instability (Spruck et al, 1999) and promotes oncogenesis (Bortner and Rosenberg, 1997), it will be important to understand the broader impact of higher Cyclin E1 levels in CD8 T cells beyond cell cycle speed.

We showed that Cyclin E1 regulation in CD8 T cells is proteasome and Nedd8-dependent, consistent with prior work in other cell types. Surprisingly, despite significantly lower Skp2 levels in CD8 T cells compared to NIH3T3 (Fig. EV2A), Cyclin E1 protein showed similar stability in the two cell types. Our results suggest that mRNA expression and translation may be crucial factors in increased Cyclin E1 protein levels in CD8 T cells. Considering that Emi1/Fbxo5 and Cyclin E1 are both E2F target genes (Caldon and Musgrove, 2010; Hsu et al, 2002), our results are consistent with generally higher E2F activity in CD8 T cells and mESCs. Further work will be required to test if E2F is the key driver (Zhu et al, 2001).

Cyclin E1 overexpression resulted in truncated G1 phases but reduced cell numbers. We attribute this to increased replication stress-induced arrest. Consistent with this idea, there was an increased frequency of G2&M phase cells in Cyclin E1 overexpressing cells. These results suggest that while increased Cyclin E1 levels promote increased cell cycle speed in CD8 T cells, additional mechanisms are likely important in preventing replication stress-induced arrest. Replication stress-induced DNA damage from underlicensed DNA results in p53 activation and p21-induced cell cycle arrest in many somatic cell types. However, it is possible therefore that the lack of sensitivity towards p21 allows CD8 T cells to bypass arrest. It will be important in the future to investigate the levels of replication stress-induced DNA damage in CD8 T cells.

### Emi1 safeguards against re-replication and promotes G1 progression in CD8 T cells

Supporting a role of APC/C in CD8 T cell cycle control, the levels of its inhibitor, Emi1, were high in CD8 T cells, and disruption of Emi1 function by CRISPR/Cas9 showed defects in G1 progression and S-phase entry in CD8 T cells. Limitations of the gene disruption approach include heterogeneity in the resulting cell population following treatment with Cas9-gRNA RNPs. CD8 T cells in which Emi1 has been deleted will be at a proliferative disadvantage, resulting in outgrowth of cells with the intact locus. This is a significant challenge in the case of rapidly proliferating CD8 T cells. However, we observed a strong re-replication phenotype (> 4 N DNA content) in >40% of cells, and other cells showed a S-phase defect (Fig. 7D). Future work examining earlier timepoints, the use of time-lapse imaging assays, and proteomics would add valuable insight into the phenotype.

In addition, it is still possible that Emi1 gene disruption results in low-level DNA damage in cells with 2 N DNA content that cannot be detected using γH2A.x immunofluorescence, and therefore further work is required. Nevertheless, these results suggest that, like mESCs, Emi1 is important to promote cell cycle progression in CD8 T cells. Moreover, it will be important to determine which proteins are affected by Emi1 deletion in CD8 T cells and mESCs to understand if increased levels of these proteins are due to Emi1-mediated inhibition of APC/C.

Our results demonstrate that CD8 T cells have specialized cell cycle control mechanisms distinct from other somatic cells to support rapid cell cycles. It will be interesting to understand how these mechanisms integrate with immune differentiation and function. Indeed, our data showed that CDK4/6 inhibiting proteins, p16 and p15, were specifically induced following IL-2 treatment, suggesting cytokine-dependent induction of key cell cycle proteins. Moreover, targeting of cell cycle control pathways is an emerging paradigm to modulate the immune system. It has been suggested that delaying G1/S promotes memory formation, as suggested previously (Lelliott et al, 2021), and can enhance CD8 T cell cytotoxic function (van Haften et al, 2026). It will also be important to understand how other chemotherapeutics that target G1/S progression, for example, recently described CDK2-specific (Yu et al, 2023; Arora et al, 2023; Patel et al, 2023) and CDK2/4/6 inhibitors (Freeman-Cook et al, 2021; Arora et al, 2023), affect immune function.

## Methods

**Table:** Reagents and tools table

Reagent/resource	Reference or source	Identifier or catalog number
**Experimental models**		
hTERT-RPE1	ATCC	CRL-4000
mESC E14Tg2a	https://www.sciencedirect.com/science/article/pii/S2211124725005108	
NIH3T3	ATCC	CRL-1658
**Recombinant DNA**		
pInducer20 Cyclin E1	Addgene/Jean Cook	109348
**Antibodies**		
m CD8a	BioLegend	100732
m CD8a	BioLegend	100724
m CD8b	Invitrogen	25-0083-82
m CD25	BioLegend	102008
m CD25	BioLegend	102016
m CD25	BioLegend	102012
m CD44	eBiosciences	48-0441-82
m CD44	Invitrogen	47-0441-82
m CD69	BioLegend	104516
m CD69	BioLegend	104514
p-H3(Ser10)	Cell Signaling	29237S
p-Rb(Ser807/811)	Cell Signaling	4277S
Cyclin E1	Cell Signaling	20808S
Cul4b	Proteintech	12916-1-AP
Alpha tubulin	Sigma	T6199
GAPDH	Santa Cruz	sc-365062
Histone H3	Abcam	ab10799
Emi1	Santa Cruz	Sc365212 AF546
Eg5	Abcam	ab283273
γH2AX	Cell Signaling	5763S
Emi1	Abcam	AB184950
Zap70	BD Biosciences	610239
Mouse IgG	Licor	926-32210
Rabbit IgG		A10043
**Oligonucleotides and other sequence-based reagents**		
Emi1/Fbxo5 ex2 gRNA, ACAGGTGCACGAATCCCCTG	This study	
Emi1/Fbxo5 ex4 gRNA, CCGGCACAATGAGTTCGTGG	This study	
Thy1 ex3 gRNA, ACAGACAAGCTGGTCAAGTG	This study	
**Chemicals, enzymes, and other reagents**		
**Software**		
Spectronaut	Biognosys	16.2.220903.53000
**Other**		

### Ethics statement

This study was approved by the Ethical Review Body at the School of Biological Sciences, University of Edinburgh. All animal experiments were approved by the University of Edinburgh Bioresearch and Veterinary Services Ethical Review body and the United Kingdom Home Office under project license P38881828 to RZ.

### Mice

Mice expressing the OT-1 TCR transgene (C57BL/6-Tg(TcraTcrb)1100Mjb/J) backcrossed to the Rag-1KO (B6.129S7-*Rag1*^*tm1Mom*^/J) background (C57BL/6 Rag-1KO OT-1 mice) were used as lymph node donors for CD8 T cells. All mice were bred and housed in individually ventilated cages under specific pathogen-free conditions at the University of Edinburgh Bioresearch and Veterinary Services (BVS) facilities. OT-1 animals express a transgenic TCR specific for the ovalbumin peptide OVA257-264 (SIINFEKL), also known as the N4 peptide.

Mice were between 5 and 12 weeks of age, and sexes were randomized for tissue donors. Mice were maintained under a 12 h light/12 h dark cycle with ad libitum access to food and water at a temperature of 19–24 ˚C and humidity of 45–65%.

### Cell lines

hTERT-RPE1 cells were purchased from ATCC and cultured in DMEM/F-12 + 10% Fetal Calf Serum (FCS). NIH3T3 cells were purchased from ATCC and cultured in DMEM + 10% FCS. Mouse ES cells (E14tg2A) were maintained in DMEM (ThermoFisher Scientific, 11960044) supplemented with 10% FCS (FCS-SA/500, LabTech), 5% KnockOut Serum Replacement (ThermoFisher Scientific, 10828028), 2 mM L-Glutamine (ThermoFisher Scientific, 25030081), 100 U/ml Penicillin–Streptomycin (ThermoFisher Scientific, 15140122), 1 mM Sodium Pyruvate (ThermoFisher Scientific, 11360070), a mixture of seven non-essential amino acids (ThermoFisher Scientific, 11140050), 0.05 mM β-mercaptoethanol (Sigma-Aldrich, M6250) and 0.1 μg/ml Leukemia Inhibitory Factor (MRC PPU Reagents and Services, DU1715). Cells were grown at 37 °C in a humidified atmosphere of 5% CO_2_. For passaging, cells were released from dishes using 0.05% Trypsin–EDTA (ThermoFisher Scientific, 25300054).

### CD8 T cell culture and stimulation

OT-1 T cells were obtained by passing tissue through 70 μM mesh filters and were cultured in IMDM medium (Sigma-Aldrich I3390) supplemented with 10% FCS, L-glutamine, 100 U/ml penicillin, 100 U/ml streptomycin, and 50 μM 2β-ME. Activation was induced by incubation with 6 nM N4 antigen peptide SIINFEKL (HY-P1771A-5mg; Cambridge Bioscience) and 1:500 αCD28 (Cat: 553294 Clone:37.51; BD Pharmigen) for up to 48 h. Activated T cells were then washed in media and returned to culture with 20 µg/ml human IL-2 (200-02; Peprotech), or 10 µg/ml IL-15 (200-15; Peprotech) with daily supplements of equivalent volume cytokine added in 24 h intervals for the duration of the culture.

### Cyclin E1 overexpression in NIH3T3 cells

HEK293T cells were transfected with the pInducer20-Cyclin E1 plasmid (Addgene #109348) and lentiviral packaging plasmids (VSV-G, PAX2) using the HYVIR transfection reagent (OZ Biosciences). Lentiviral supernatant was then concentrated using Lenti-X (Takara Bio). Concentrated lentiviral supernatant was then used to transduce NIH3T3 cells. Transduced cells were then selected for the neomycin resistance marker gene using G418 to produce the NIH3T3 CyclinE1^OE^ cell line. Doxycycline was added at the indicated concentrations to induce Cyclin E1 expression.

### Flow cytometry

Cells were removed from culture and stained with Live/Dead fixable near-IR Dead cells stain kit (L34975; Thermofisher) as per the manufacturer’s instructions.

Surface staining was done in FACS buffer (0.1% BSA (Fisher bioreagents; BP9702-100), 0.01% Sodium Azide (S8032; Sigma-Aldrich), PBS (D1408-500ml; Sigma-Aldrich) with antibodies at the appropriate concentration for 20 min at 4 °c.

Cells were washed in PBS and resuspended and fixed in 2% Formaldehyde (28908; Thermofisher) for 30 min at room temperature in constant rotation.

Cells requiring intracellular staining were resuspended in <10 µl FACS buffer. Once resuspended, they were permeabilized by 500 µl ice cold 90% MeOH (M/4000/PC17; Fisher bioreagents), which was added dropwise with gentle vortexing to the cells and stored at −20 °C.

Prior to the intracellular stain, the cells were washed twice with FACS buffer and then incubated with antibodies at the appropriate concentration for 1 h at 4 °C. The cells were then washed and acquired in FACs buffer or FACs buffer with 1 µg/ml Hoechst 33342.

Data were acquired using either a MACSQuant Analyzer (Miltenyi), Novocyte (Agilent), or a Fortessa (BD Biosciences).

Formaldehyde-fixed, methanol-permeabilized CD8 T cells were stained with anti-phosphoRb (S807/S811) and Hoechst and separated into G1^2N,pRb-^, G1^2N,pRb+^, S^2N-4N,pRb+^, and G2&M^4N,pRb+^ phase fractions using a FACSAria IIu sorter (BD Biosciences). Pilot pRb staining performed on mESCs and NIH3T3 showed the absence of a pRb-negative population. Therefore, for sorting, no pRb staining was performed. Formaldehyde-fixed, methanol-permeabilized mESCs and NIH3T3 were stained with EdU and Hoechst and separated into G1^2N,EdU-^, S^2N-4N,EdU+^, and G2&M^4N,EdU-^ phase fractions using a MA900 sorter (Sony Biosciences).

### EdU click reaction

Cells were stained for EdU with the use of the Click-IT EdU imaging kit (C10337; Invitrogen) as per the manufacturer’s instructions, with some modifications. Cells in culture were pulsed with 1 µM EdU for 30 min prior to removal of culture and processed for flow cytometry as above. Prior to intracellular staining, the cells were incubated with the click reaction mix, swapping out the azide-488 that came with the kit for azide-AF594 (CLK-1295-1; Jena Bioscience).

### CellTraceViolet proliferation assay

Naive cells were washed with PBS and incubated at 37 °C for 20 min with 5 µM Cell Trace Violet (C34571A; Invitrogen) at a volume of 1 ml per 1e7 cells. Following incubation, the cells were quenched with 2× volume of media and incubated at 37 °C for 5 min.

### Immunoblot analysis

Cell pellets were lysed using 2% SDS + protease inhibitor cocktail (P8340ML; Sigma-Aldrich), incubating at 65 °C for 5 min, 1 µl Benzonase (70664-3; Sigma-Aldrich) was added and incubated for a further 15 min. The lysates were then sonicated with a Ultrasonic Processor (UP200ST; Hielscher) at full power for two cycles of 20 s on 30 s off. Protein content for lysates were then assessed using Micro BCA Protein Assay Kit (23235; Thermofisher) as per the manufacturer’s instructions.

Lysates were incubated with NuPage LDS Sample Buffer (NP0007; Invitrogen) and incubated at 95 °C for 5 min and loaded into NuPage 4%-12% Bis-Tris gradient MOPS gel (NP0323BOX; Invitrogen) in MOPS SDS Running Buffer (NP0001; Invitrogen) and run at 70 volts for 15 min and then 140 volts for 75 min. The gel was when blotted on to an Immobilon-FL PVDF membrane (IPFL00010; Millipore Sigma) submerged in transfer buffer (24 mM Tris Base (BP152-1; Fisher Bioreagents), 192 mM Glycine (G8898-1KG; Sigma-Aldrich), 20% Ethanol (20821.330; VWR)) at 120 volts for 105 min, and then left to block in Intercept Blocking buffer (927-70001; Licor) for 1 h or overnight.

The membrane was stained in primary antibody diluted in blocking buffer + 0.1% Tween 20 (H5151; Promega) for 4 h or overnight, and the secondary antibody diluted in blocking buffer 0.1% Tween 20, and 0.01% SDS. The membrane was washed following both stains in PBS + 0.1% Tween.

The membrane was imaged using a Licor Odyssey CLx (Licor) and analyzed using ImageJ and ImageStudio (Licor).

### Proteomics sample preparation

Sorted cells undergoing mass spec analysis were washed and resuspended in triethylammonium bicarbonate (TEAB) digestion buffer (100 nM TEAB, 50 µM CaCL_2_, 50 µM MgCL_2_), incubated with 1 µl Benzonase at 37 C for 15-30 min, and then digested using the in-cell digestion protocol (Kelly et al, 2022) using Pierce Trypsin Protease (90057; Thermo Fisher) overnight at a ratio of 1 µg trypsin per 20 µg of protein. Peptides were acidified in either 1% trifluoroacetic acid (TFA). Fragments of C18 silica membrane were placed in 200 µl tips, wetted with 100% acetonitrile (MeCN) (A955-1; Fisher Chemicals) and primed with 1% TFA/formic acid (10596814; Thermo Fisher). For samples in excess of 100 µg, higher capacity Sep-Pak Vac tC18 cartridges (WAT036790; Waters) were used instead. The sample was loaded onto pre-conditioned columns, washed with 0.1% TFA, and then eluted into fresh tubes with 70% MeCN diluted in either 0.1% TFA, or 0.1% formic acid.

### Mass spectrometry

For data-independent analysis (DIA): Peptides (equivalent of 1 µg) were injected onto a C18 reverse-phase chromatography system (UltiMate 3000 RSLC nano, Thermo Scientific) and electrosprayed into an Orbitrap Exploris 480 Mass Spectrometer (Thermo Fisher). For liquid chromatography, the following buffers were used: buffer A (0.1% formic acid in Milli-Q water (v/v)) and buffer B (80% acetonitrile and 0.1% formic acid in Milli-Q water (v/v). Samples were loaded at 10 μL/min onto a trap column (100 μm × 2 cm, PepMap nanoViper C18 column, 5 μm, 100 Å, Thermo Scientific) equilibrated in 0.1% trifluoroacetic acid (TFA). The trap column was washed for 3 min at the same flow rate with 0.1% TFA, then switched in-line with a Thermo Scientific, resolving C18 column (75 μm ×  50 cm, PepMap RSLC C18 column, 2 μm, 100 Å). Peptides were eluted from the column at a constant flow rate of 300 nl/min with a linear gradient from 3% buffer B to 6% buffer B in 5 min, then from 6% buffer B to 35% buffer B in 115 min, and finally from 35% buffer B to 80% buffer B within 7 min. The column was then washed with 80% buffer B for 4 min. Two blanks were run between each sample to reduce carryover. The column was kept at a constant temperature of 50 °C.

The data was acquired using an easy spray source operated in positive mode with spray voltage at 2.40 kV, and the ion transfer tube temperature at 250 °C. The MS was operated in DIA mode. A scan cycle comprised a full MS scan (*m/z* range from 350 to 1650), with RF lens at 40%, AGC target set to custom, normalized AGC target at 300%, maximum injection time mode set to custom, maximum injection time at 20 ms, microscan set to 1, and source fragmentation disabled. The MS survey scan was followed by MS/MS DIA scan events using the following parameters: multiplex ions set to false, collision energy mode set to stepped, collision energy type set to normalized, HCD collision energies set to 25.5, 27, and 30%, orbitrap resolution 30,000, first mass 200. Data for both MS scan and MS/MS DIA scan events were acquired in profile mode.

### Proteomic data analysis

RAW data files from DIA experiments were processed using DIA-NN (version 1.8.1). The default settings for the library-free workflow were used with oxidation of methionine set as a variable modification. The database used was M.musculus downloaded from uniprot.org on 2024-07-24.

The output from DIA-NN was then processed in R (4.0.3). Protein abundances were normalized for technical differences in peptide loading by dividing each protein intensity by the sum of all protein intensities and multiplying the result by 1e6 (parts per million, ppm). A ppm is thus equal to one part per million intensity units detected in the sample. G1 intensities in the CD8 T cell dataset were corrected for S-phase contamination by calculating the %EdU+ cells in the 2N DNA content gate (e.g., Fig. 1C). This fraction was then multiplied by the S-phase intensity to estimate the fractional intensity originating from S-phase contamination and subtracted from the intensity measured in the G1 fraction. GO term analysis was done by exporting a list of proteins from the dataset and retrieving the Gene Ontology (GO) terms from Uniprot.org. Further analysis of GO terms was done utilizing the DAVID Bioinformatics resources (https://david.ncifcrf.gov/) (Huang et al, 2009). To identify cell cycle-regulated proteins in all three cell lines, first missing values were imputed using Gaussian-based distributions for noise (Harris et al, 2023). ANOVA and Tukey’s multiple comparison test were performed to test for significant differences in protein abundance between cell cycle phases. Fold changes were calculated for all pairwise contrasts. This statistical analysis was repeated separately for each cell type. Proteins were then deemed significantly changing if they met the following filtering criteria: p < 0.05 and fold change >2. The *P* values are unadjusted for multiple hypothesis testing. These proteins were then clustered using hierarchical clustering using the “complete” agglomeration algorithm implemented in R. The cluster that showed cell cycle-regulated protein abundance in all three cell lines was used for further analysis.

### CRISPR

Target PAM sites for CRISPR were identified using ChopChop (https://chopchop.cbu.uib.no/) with default settings (Montague et al, 2014). Candidates were selected by for their high GC content, low number of mismatch pairs, and high efficiency. The following gRNAs were synthesized to disrupt the Emi1/Fbxo5 and Thy1 genes (Integrated DNA Technologies): ACAGGTGCACGAATCCCCTG (Emi1/Fbxo5 gRNA targeting exon 2), CCGGCACAATGAGTTCGTGG (Emi1/Fbxo5 gRNA targeting exon 4), ACAGACAAGCTGGTCAAGTG (Thy1 gRNA targeting exon 3).

gRNA-Cas9 transfection was performed using Neon Transfection 100 µl kit (MPK10096; Invitrogen). gRNA was incubated with tracrRNA (B00817; IDT) in Nuclease free duplex buffer (1072570; IDT) for 5 min at 98 C. Samples were then allowed to cool down before incubating with the TrueCast Cas9 protein V2 (A36499; Thermo Fisher) and Resuspension Buffer T for 20 min at 37 °C. T cells were pelleted at 300 G for 5 min and resuspended in Resuspension Buffer T at a concentration of 1.2e6 cells/100 μL. 1.2e6 cells were mixed with the assembled Cas9 complex, and electroporation was carried out utilizing the Neon 100 µl Tips within a solution of Electrolytic Buffer E2 provided by the kit. Electroporation was induced using the Neon Transfection System (Invitrogen) with a program of 3 pulses of 10 ms 1600 V for Activated T cells.

### TIDE analysis

Primers were designed to assess CRISPR KO efficiency via TIDE (Brinkman et al, 2014). The criteria for their design were: (1) the primer target sequence must be between 250 and 450 bp from the Cas9 cut site, and (2) that the PCR product size must be ≥500 bp long. These criteria were implemented in the Primer-BLAST webtool (https://www.ncbi.nlm.nih.gov/tools/primer-blast/), and primers were chosen based on the fewest number of off-target matches.

PCR mixes were created using the Phusion High-Fidelity DNA Polymerase Reagents (M0530L; New England Biolabs). Samples were spun down and removed of liquid before being lysed with PCR Lysis buffer (0.1% SDS, 0.05% Tween, 400 µg/ml Proteinase K (25530-015; Life Technologies). PCR was then conducted with the following settings on a T100 Thermo Cycler (Biorad). 98 C for 1 min, 98 °C for 15 s, optimal temperature (between 62 and 72 °C) for 15 s, 72 °C for 45 s, cycle 34 times, then 72 °C for 10 min, and hold at 12 °C. A fraction of the PCR product was run through a 1.5% Agarose gel made from UltraPure Agarose (16500-500; Invitrogen) in TAE buffer. Electrophoresis was performed at 120 V for 20 min and imaged on a Licor D-Digit. The remaining product was then purified using the Monarch PCR & DNA Cleanup Kit (T1030L; New England Biolabs). The purified product was then quantified with a Qubit 3.0 (Invitrogen) and sent to the MRC Protein Phosphorylation and Ubiquitylation Unit in Dundee (https://dnaseq.co.uk/) for sequencing. The returned sequences were then analyzed using the TIDE analysis webtool (https://tide.nki.nl/) (Brinkman et al, 2014).

### Measurement of Cyclin E1 stability

To prepare OT-1 T cell cultures, first single-cell lymphocyte suspensions were obtained by mashing lymph nodes (brachial, axillary, inguinal, and superficial cervical) through a 70-μm strainer directly into complete RPMI. For generation of OT-1 CTL, T cell receptor stimulation was performed using the cognate antigen N4 (OVA 257-264, SIINFEKL) at 10 ng/ml in the presence of IL-2 (20 ng/ml) (Proleukin). After 48 h activation, cells were washed in complete RPMI to remove activation conditions and resuspended in pre-warmed complete RPMI medium with IL-2 at 20 ng/ml. Cells were split daily to a density of 0.3 × 10^6^ cells/ml cells/ml into fresh warm complete RPMI with IL-2 for a further 3 days.

Cells were incubated at 37 °C at 5% CO_2_ in complete culture medium comprised of RPMI 1640 Medium with L-glutamine (Gibco 21875091) supplemented with 10% FBS (Gibco 10270106), 100 U/ml penicillin/streptomycin (Gibco 11548876), and 50 μM 2-mercaptoethanol (Gibco 31350010).

## Supplementary information

Peer Review File

Dataset EV1

Dataset EV2

Dataset EV3

Dataset EV4

Source data Fig. 1

Source data Fig. 5

Source data Fig. 7

Figure EV2 Source Data

Figure EV3 Source Data

Figure EV4 Source Data

Expanded View Figures

## Data Availability

The mass spectrometry-based proteomics data have been deposited to the ProteomeXchange Consortium via the PRIDE (Perez-Riverol et al, 2025) partner repository with the dataset identifier PXD077442. The source data of this paper are collected in the following database record: biostudies:S-SCDT-10_1038-S44319-026-00830-4.
