# Supplementary material for: CD8 T lymphocytes deploy embryonic cell cycle control mechanisms for rapid cell proliferation
Source: EMBO Rep. 2026 Jun 18;27(15):4272–98. doi: 10.1038/s44319-026-00830-4 (PMC13458459; doi:10.1038/s44319-026-00830-4)
Supplement: Supplementary file 1 [file 44319_2026_830_MOESM12_ESM.pdf]

Expanded View Figures

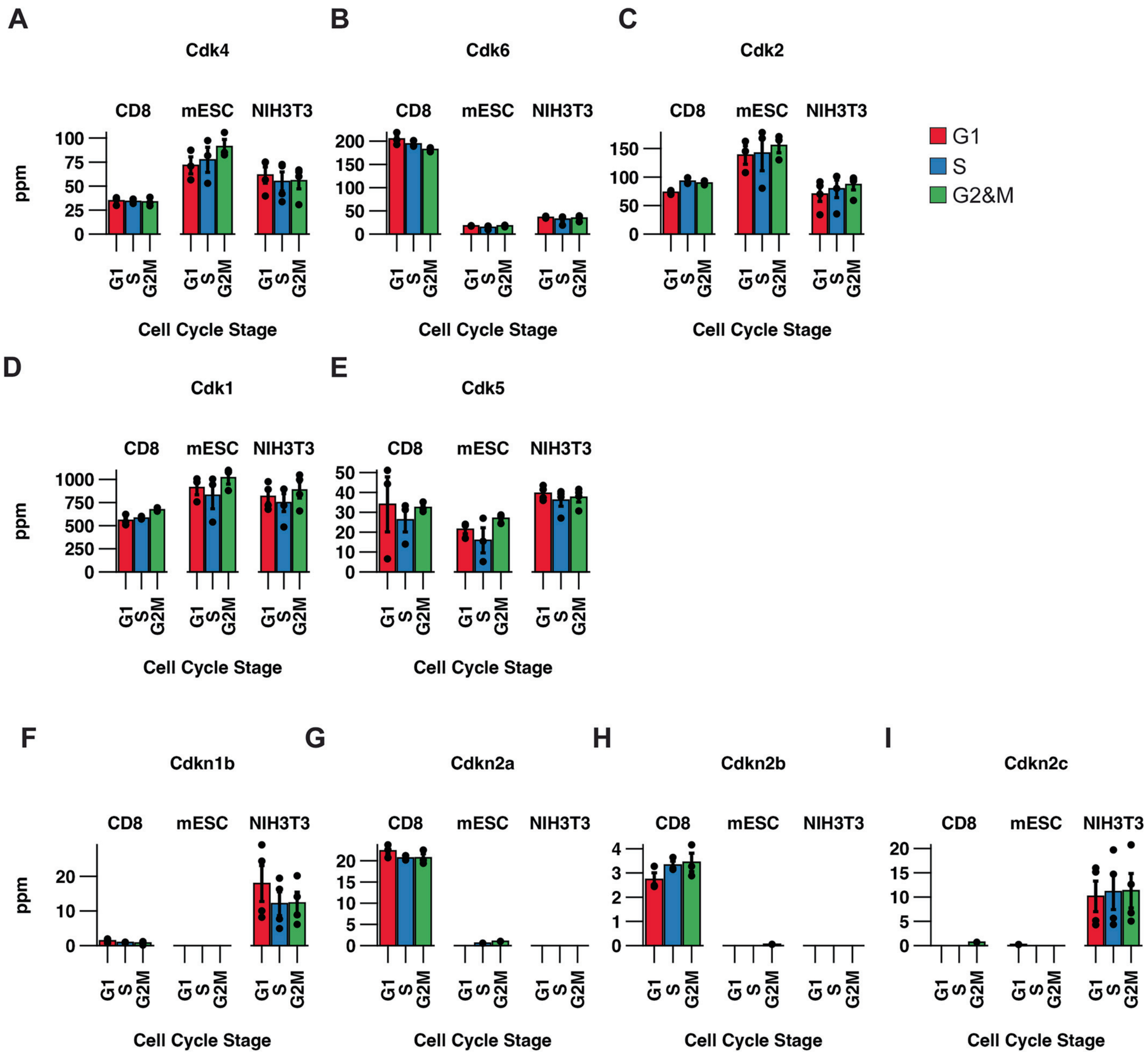

Figure EV1. CDK and CDK inhibitor abundances.

Protein intensities (ppm) for Cdk4 (**A**), Cdk6 (**B**), Cdk2 (**C**), Cdk1 (**D**), Cdk5 (**E**), Cdkn1b (p27) (**F**), Cdkn2a (p16) (**G**), Cdkn2b (p15) (**H**), and Cdkn2c (p18) (**I**). Individual points are from distinct animals (CD8 T cells) or different passages (mESC, NIH3T3). The bar height indicates the mean, and error bars indicate standard error of the mean ($n = 3$–4). For conditions in which a protein was not detected, no bar is shown.

 © The Author(s)

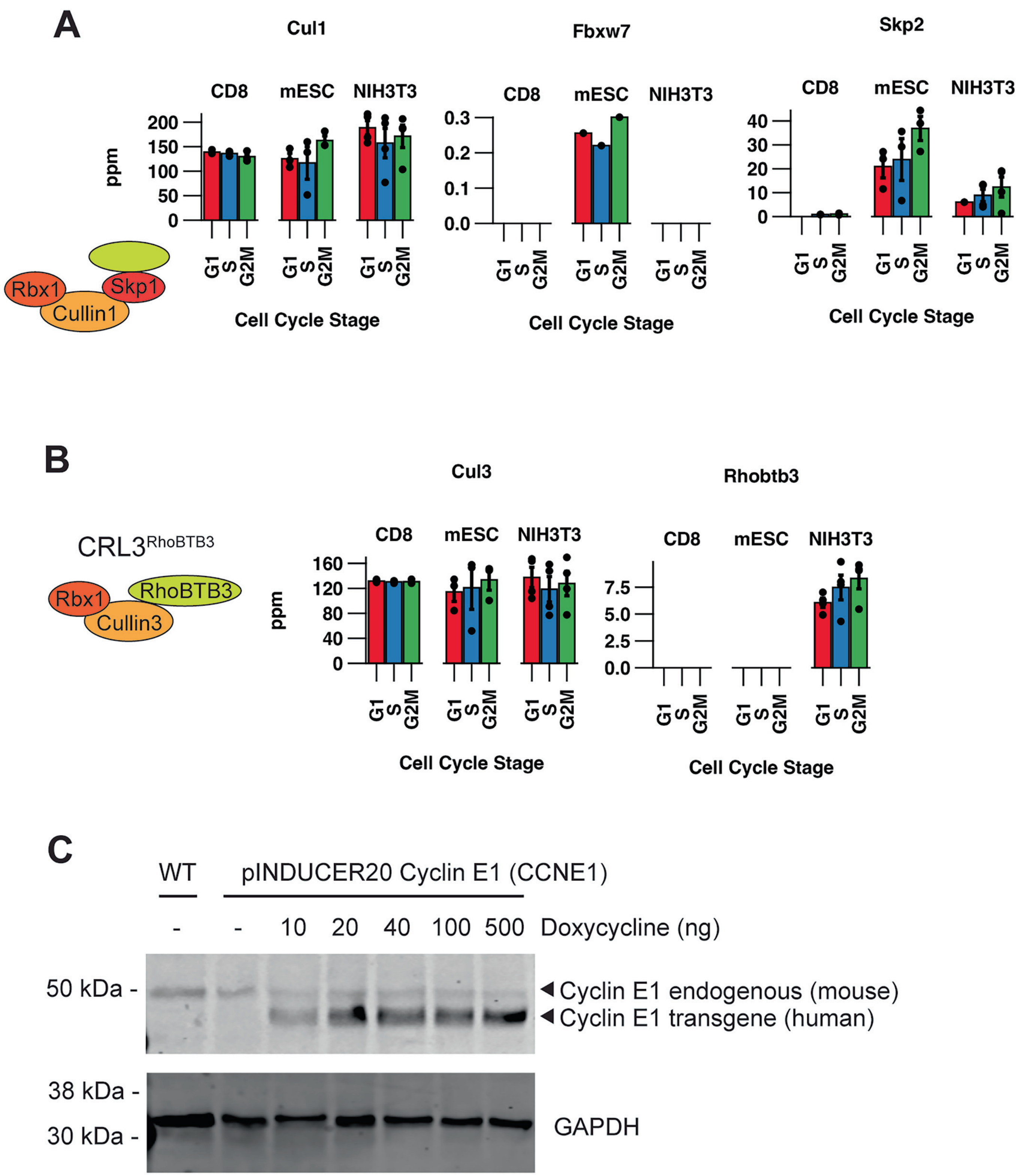

Figure EV2. Cyclin E1 regulation and overexpression.

Protein intensities (ppm) for Cul1, Fbxw7, and Skp2 (**A**), and Cul3 and Rhobtb3 (**B**). Individual points are from distinct animals (CD8 T cells) or different passages (mESC, NIH3T3). The bar height indicates the mean, and error bars indicate standard error of the mean ($n = 3$–4). For conditions in which a protein was not detected, no bar is shown. (**C**) Immunoblot showing expression of Cyclin E1 transgene following doxycycline treatment of NIH3T3 CCNE1^{OE} cells. Source data are available online for this figure.

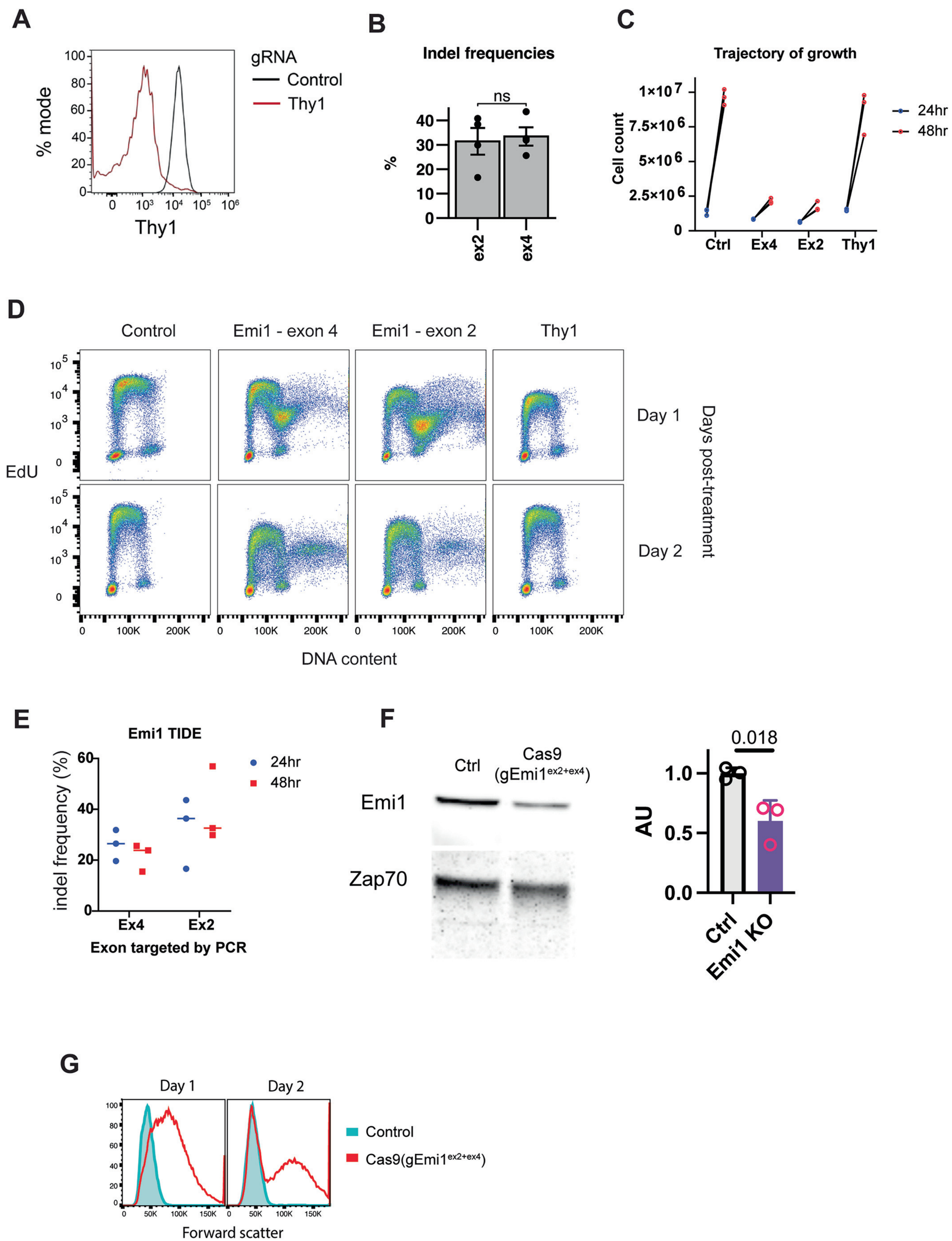
A
% mode
Thy1
gRNA
Control
Thy1
B
Indel frequencies
ns
%
ex2
ex4
C
Trajectory of growth
Cell count
Ctrl
Ex4
Ex2
Thy1
24hr
48hr
D
Control
Emi1 - exon 4
Emi1 - exon 2
Thy1
EdU
Day 1
Day 2
Days post-treatment
DNA content
E
Emi1 TIDE
indel frequency (%)
24hr
48hr
Ex4
Ex2
Exon targeted by PCR
F
Ctrl
Cas9 (gEmi1ex2+ex4)
Emi1
Zap70
0.018
AU
Ctrl
Emi1 KO
G
Day 1
Day 2
Forward scatter
Control
Cas9(gEmi1ex2+ex4)

 © The Author(s)

Figure EV3. Emi1 promotes S-phase entry and safeguards against re-replication.

(**A**) CD8 T cells treated with Cas9(gThy1^{ex3}), stained with anti-Thy1 antibody, and analyzed by flow cytometry. (**B**) CD8 T cells treated with either Cas9(gEmi1^{ex2}) or Cas9(gEmi1^{ex4}) and cultured for 24 h were harvested for genomic DNA. Two gRNAs were tested, which target exon 4 (Ex4) and exon 2 (Ex2) of the Emi1/Fbxo5 locus. Indel frequencies were measured by PCR and TIDE analysis. ($n = 3$ individual animals), statistical significance assessed using *t* test. (**C**) Cell counts following control (electroporation with no Cas9), Cas9(gEmi1^{ex2}), Cas9(gEmi1^{ex4}), or Cas9(gThy1^{ex3}) treatment and culturing cells for 24 and 48 h. ($n = 3$ individual animals) (**D**) Cell cycle analysis of cells as in (**C**). (**E**) TIDE analysis of cells treated with Cas9(gEmi1$^{ex2+ex4}$). ($n = 3$ individual animals), (**F**) Immunoblot analysis of extracts from control and Cas9(gEmi1$^{ex2+ex4}$) treated cells, probing for Emi1 protein. Zap70 was probed to assess loading. ($n = 3$ individual animals), statistical significance assessed using *t* test. (**G**) Forward scatter measurements of control and Cas9(gEmi1$^{ex2+ex4}$) treated cells. Forward scatter is indicative of cell size. Source data are available online for this figure.

© The Author(s)

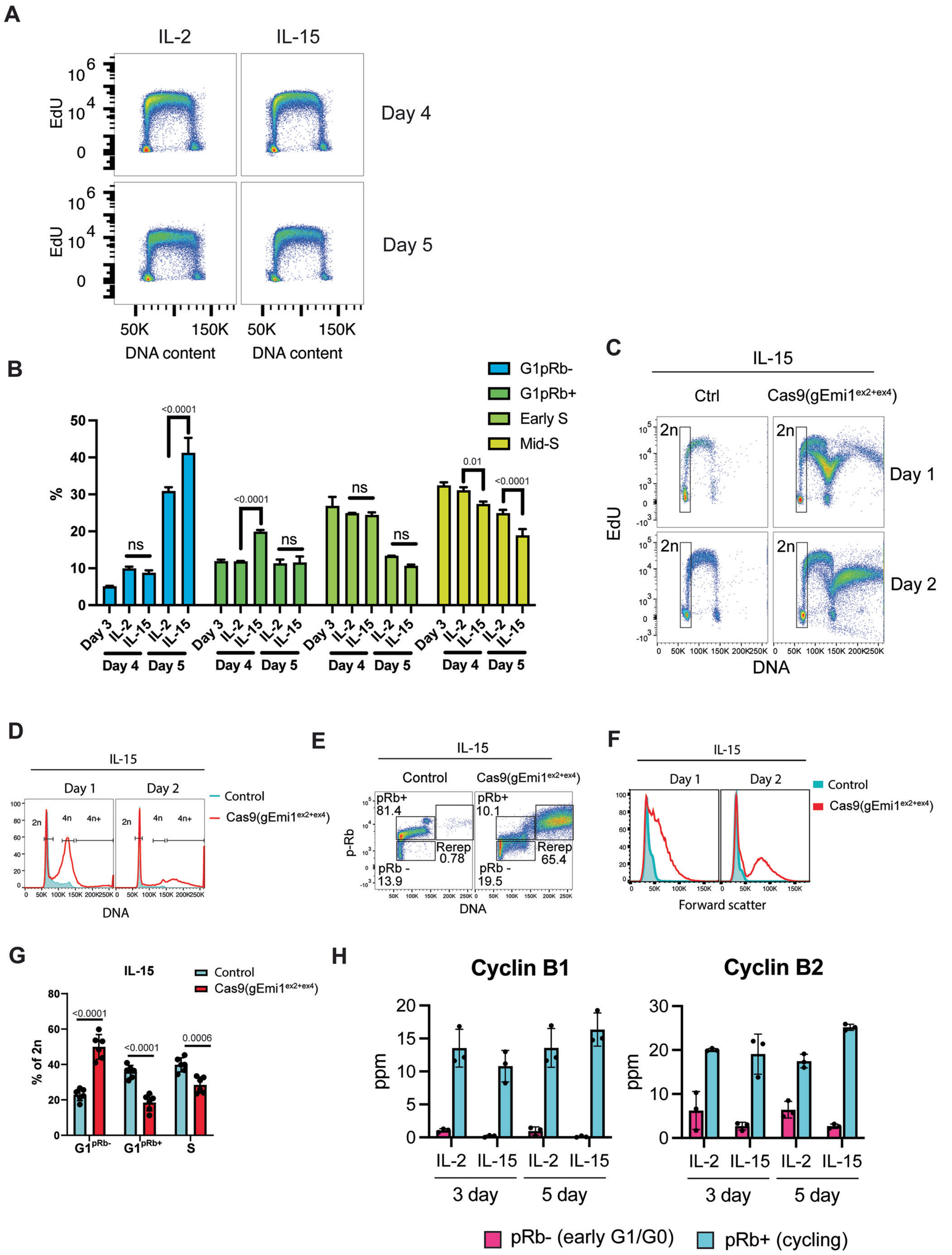
A
IL-2
IL-15
Day 4
Day 5
EdU
DNA content
B
G1pRb-
G1pRb+
Early S
Mid-S
%
ns
<0.0001
0.01
Day 3
IL-2
IL-15
Day 4
Day 5
C
IL-15
Ctrl
Cas9(gEmi1ex2+ex4)
2n
Day 1
Day 2
EdU
DNA
D
IL-15
Day 1
Day 2
2n
4n
4n+
Control
Cas9(gEmi1ex2+ex4)
DNA
E
IL-15
Control
Cas9(gEmi1ex2+ex4)
p-Rb
pRb+ 81.4
Rerep 0.78
pRb - 13.9
pRb+ 10.1
Rerep 65.4
pRb - 19.5
DNA
F
IL-15
Day 1
Day 2
Control
Cas9(gEmi1ex2+ex4)
Forward scatter
G
IL-15
Control
Cas9(gEmi1ex2+ex4)
% of 2n
<0.0001
0.0006
G1pRb-
G1pRb+
S
H
Cyclin B1
Cyclin B2
ppm
IL-2
IL-15
3 day
5 day
pRb- (early G1/G0)
pRb+ (cycling)

 © The Author(s)

Figure EV4. Role of Emi1 in memory-like CD8 T cells.

(**A**) CD8 T cells were activated for two days and then cultured either in IL-2 or IL-15 for an additional 2 or 3 days. Cell cycle distribution was measured using phosphorylated Rb, EdU, and DNA content. Representative cell cycle profiles are shown. (**B**) Calculated frequencies from data shown in (**A**). Total time post activation is shown on the *x* axis. (**C**) EdU incorporation of CD8 T cells activated at day 0, treated with Cas9(gEmi1$^{ex2+ex4}$) or a blank control on day 2, cultured IL-15, and cultured for an additional one or two days prior to harvest for analysis. (**D**) DNA content histograms of cells from (**C**); (**E**) Rb phosphorylation of cells from (**C**). (**F**) Forward scatter measurements by flow cytometry of cells from (**C**); (**G**) quantitation of early G1/G0 (G1^{pRb-}), late G1 (G1^{pRb+}), and early S-phase cells of cells from (**C**). Statistical significance was determined by unpaired *t* tests, $n = 6$ mice from two independent cohorts (**A**–**F**), $n = 3$ mice (**G**). (**H**) Protein abundance (ppm) of Cyclin B1 and Cyclin B2 comparing IL-2 and IL-15 treated cells ($n = 3$). Source data are available online for this figure.

© The Author(s)
