# Supplementary material for: CD8 T lymphocytes deploy embryonic cell cycle control mechanisms for rapid cell proliferation
Source: EMBO Rep. 2026 Jun 18;27(15):4272–98. doi: 10.1038/s44319-026-00830-4 (PMC13458459; doi:10.1038/s44319-026-00830-4)
Supplement: Supplementary file 2 [file 44319_2026_830_MOESM1_ESM.pdf]

EMBO reports

CD8 T lymphocytes deploy embryonic cell cycle control mechanisms for rapid cell proliferation

David Lewis, Ananya Kar, Alice Savage, Van Kelly, David Wright, Devin Tan, Mary Tozer, Christina Rollings, Doreen A. Cantrell, Rose Zamoyska, and Tony Ly

Corresponding author(s): Tony Ly (t.ly@dundee.ac.uk)

Review Timeline:

Transfer Date:	4th Mar 26
Editorial Decision:	8th Apr 26
Revision Received:	29th Apr 26
Editorial Decision:	13th May 26
Revision Received:	20th May 26
Accepted:	30th May 26

Editor: Achim Breiling

Transaction Report: This manuscript was transferred to EMBO Reports following peer review at Review Commons.

Review COMMONS

(Note: With the exception of the correction of typographical or spelling errors that could be a source of ambiguity, letters and reports are not edited. Depending on transfer agreements, referee reports obtained elsewhere may or may not be included in this compilation. Referee reports are anonymous unless the Referee chooses to sign their reports.)

Review #1

1. Evidence, reproducibility and clarity:

Evidence, reproducibility and clarity (Required)

In this study by Lewis and colleagues, the authors use highly quantitative proteomics to compare cell cycle patterns in T lymphocytes to mouse embryonic stem cells and differentiated fibroblasts. The work is significant because the cell cycle field has been dominated by only a few paradigms based on model systems such as yeasts, cancer-derived cell lines, and fibroblasts. The different cell cycles in other cell types have not been thoroughly investigated because they are less experimentally tractable. Prior studies have drawn conclusions about embryonic stem cells that turned out later to be primarily from attempts to induce synchronization prior to analysis. Here, the authors apply their previously-described method for detecting proteins according to cell cycle phase by flow-sorting and then mass spectrometry. They find expected cell cycle fluctuations in the three cell lines, but also a few unexpected differences. Primarily, cyclin E protein appears to be expressed at higher levels in the fast-proliferating mESCs and CD8 cells compared to NIH3TS and to be less cell cycle-regulated. The mechanism and functional relevance for this difference are not considered.
The authors chose to focus several experiments on the APC/C ubiquitin ligase inhibitor, Emi1, which they find appropriately cell cycle-regulated in CD8 and mESCs but expressed at higher levels in CD8 cells. They show that CD8 differentiation is associated with cell cycle changes and that perturbing the cell cycle in CD8 cells affects their differentiation (which is not surprising). Altogether this study provides an excellent resource for the field from the detailed high-quality proteomics data sets (though see point #8). On the other hand, the experiments with EMI1-KO and differentiating cells seem largely unrelated to the cell cycle analysis itself; these results may be mostly indirect consequences of cell cycle perturbation rather than specific to EMI1 function. This is a very thorough, rigorous, and valuable proteomics characterization of cell cycle differences, but the choice of follow-up functional experiments isn't logical in my opinion.

Major points:

1. It is not clear how the quantification accounts for differences in cell size or nuclear/cytoplasmic fractions among the different cell types. For example, ESCs are typically small with relatively less cytoplasm. Since a great many cell cycle proteins are nuclear, normalizing to total protein analyzed and not cell number could make these

proteins appear more abundant. In other words, does 1 ug of protein from mESCs represent the same number of cells or nuclei as 1 ug of protein from NIH3T3?
2. The motivation for the project was given as wanting to understand if fast-cycling CD8 T cells resemble fast-cycling mESCs, which the authors show they partially do. The molecular phenotype in common is cyclin E abundance, but we are left without an understanding for how that occurs. Are there differences in expression of the E3 ligase for cyclin E?
3. We also don't learn if the constitutive cyclin E is why cells cycle quickly because no experiments to change cyclin E are provided.
4. In an unexpected turn for the project, the authors generated inducibly Emi1-null CD8 T cells and analyzed them for replication, re-replication, and phosphorylated Rb. This section is fine, but the rationale isn't obvious, particularly if constitutive cyclin E (not an APC/C substrate) is the proposed driver of fast proliferation. Emi1 isn't the general reason for fast proliferation if it is only high during G1 for CD8 and not mESCs. Emi1 loss has also been known to promote re-replication for quite a few years, a finding the authors cite. The replication-dependent DNA damage and cell cycle arrest are both well-known outcomes from re-replication. So, although the data are of good quality, I'm not sure that showing Emi1 has the same role in CD8 cells as what was reported in other cells adds much to the field. The authors could choose to save Figure 6 for a more APC/C-focused study and use EMI1 as a general cell proliferation marker in Figure 7.
5. What is the cell cycle distribution of the IL-15 and IL-2 differentiated cells? If they are different, then some of the protein abundance changes to regulated proteins such as p21 might be a symptom of cell cycle differences rather than differentiation. It seems IL-15 is more cell cycle promoting form Figure S3, but that isn't clear from the assays presented.
6. The change in the CD62L marker is argued to be unrelated to cell cycle and somehow more directly downstream of Emi1. The connection here is tenuous, in part because there's no comparison to a similar cell cycle perturbation independent of Emi1. How would Emi1 modulate CD62L if CDC62L does not show evidence of being an APC/C substrate?
7. As presented, Emi1 has a unique effect on the immune phenotype. It is also possible that many cell cycle perturbations would similarly affect differentiation and not only Emi1. Can the authors distinguish Emi1-specific effects from more common cell cycle perturbation effects? Alternatively, interpreting the EMI1-KO data more generally could avoid confusion.
8. The authors should provide legends to the supplementary tables in the text and/or the tables themselves. Several column headers in the tables are not defined.

Minor points:

- a. Figure 4g is titled Cdkn2a (which would be p16) but the legend says its Cdkn1a (p21). The p21 protein was undetected in NIH3T3? One would expect it to be in the G1 cells at least.
- b. Line 56: cyclin E is not an APC/C substrate as implied by the text. If Skp2 is a bona fide substrate (not sure that's true), then APC/C activity would have opposite effects on Cyclin E vs Cyclin A.
- c. What is the red arrow in Figure 6C?
- d. Figure 7b is protein copy number whereas all other graphs are for ppm. The legend on line 686 has "cite immPRES" but this citation didn't make it into the submitted manuscript.

2. Significance:

Significance (Required)

The work is significant because the cell cycle field has been dominated by only a few paradigms based on model systems such as yeasts, cancer-derived cell lines, and fibroblasts. The different cell cycles in other cell types have not been thoroughly investigated because they are less experimentally tractable. Prior studies have drawn conclusions about embryonic stem cells that turned out later to be primarily from attempts to induce synchronization prior to analysis. This is a very thorough, rigorous, and valuable proteomics characterization of cell cycle differences, but the choice of follow-up functional experiments isn't logical in my opinion.

3. How much time do you estimate the authors will need to complete the suggested revisions:

Estimated time to Complete Revisions (Required)

(Decision Recommendation)

Between 3 and 6 months

4. *Review Commons* values the work of reviewers and encourages them to get credit for their work. Select 'Yes' below to register your reviewing activity at Web of Science Reviewer Recognition Service (formerly Publons); note that the content of your review will not be visible on Web of Science.

Web of Science Reviewer Recognition

No

Review #2

1. Evidence, reproducibility and clarity:

Evidence, reproducibility and clarity (Required)

Summary

This study uses proteomics as an approach to understand regulatory mechanisms contributing to a short cell division cycle in CD8 T cells. While the cell cycle machinery has been studied in mouse embryonic stem cells (mESCs) and these discoveries have provided a framework for understanding atypically short cell cycles, whether those mechanisms are conserved in other cell types that undergo a short cycle has not been clear. Analysis of the cellular proteome was performed at three different cell-cycle stages (G1, S, G2/M) and across three cell lines (CD8 T cells and mESCs and fibroblast 3T3 cells as comparators for short and normal cycles, respectively). An important innovation here is the application of a PRIMMUS protocol, which uses markers of cell cycle stage and sorting rather than treatments, such as inhibitors, that can have unwanted perturbations. The authors find both similarities and differences between CD8 T cells and mESCs. Of particular interest, CycE levels are constitutively high in both cell types with a short cycle compared to fibroblast cells, but APC/C substrates are uniquely lower during G1 in CD8 T cells. Using CRISPR knock-out, the APC/C inhibitor Emi1 is then implicated as a key regulator of G1/S control in CD8 T cells as well as phenotypic fate.

Major Comments:

Overall, the conclusions are fairly made from the data. In particular, the proteomics data and analysis are of high technical quality. The data and methods are clearly presented in a manner that could be reproduced, and experiments are, with one exception noted gere, performed with appropriate replicates and statistical analysis. I have only two major comments.

1. From the description of the CRISPR methods and results, the origin of the Emi 1KO cells used in all the experiments is unclear. Only one Emi1 KO sample or experiment is shown along with control in each panel. A number of questions should be answered in order to clarify. Was this sample originated from the total cells grown out of the gRNA treatment (and on which the TIDE analysis was performed)? If so, which exon was the gRNA targeting in the cells used in subsequent experiments? Were single clones ever grown? Ideally, experiments should be performed with multiple single clonal lines, and at least one from

each exon used, if possible. Alternatively, a population generated from each of the two guids should be used in all the experiments as a proper biological replicate.

It may be that homozygous deletion of Emi1 is not possible because of the effects on proliferation. If so, the authors should comment on this along with why the protein level in Sup. Fig. 2b is still so high in the KO line. Considering the inefficiency of the overall KO, it is especially important to understand Emi1 expression throughout the polyclonal population to interpret the results. Are there some cells expressing normal levels and some cells expressing no Emi1? If monoclonal, are the observed effects from haploinsufficiency? More description of the method and caveats to the interpretation of results are needed here, and results would be made more robust if more than one clone or gRNA treatment were used. It would be expected this may take 2-3 months with reasonable resources to perform this additional analysis, if not done already.
2. OPTIONAL. One shortcoming of the manuscript is the lack of insights into why knock out of Emi1 leads to G1 arrest. Follow up experiments on the APC/C substrates in Fig. S1b or analysis of proteins that are upregulated or downregulated by Emi KO would increase the significance of the manuscript. These experiments may be time consuming and could be considered beyond the scope of this study, but they would increase the impact of the study.

Minor comments:

1. Some brief aspects of the methods could be added to the description of results to help improve clarity in a few places. For example, the threshold for including the 121 proteins in Fig. 2b and what antibodies were used for staining in the PRIMMUS approach would be important enough that it should be stated in results with no need to look up detailed method.
2. It should be clarified that in most cases, use of the term "abundance" in comparing protein levels is actually a relative abundance for a specific cell type. For example in the following sentence, relative and not absolute abundances are being compared : "In mESC, there was higher abundance in G1 phase compared to CD8 T cells and NIH3T3 (Fig.136 2d)." Clarifying upon first use of the term abundance would be sufficient.
3. In some of the protein abundance panels there does not appear to be any mark in the graph, which is consistent with there being no observation of protein. This absence of any observation should be made explicit in the figure captions.
4. It should be clarified with its first use (line 264) that "pRb" means phosphorylated Rb.

2. Significance:

Significance (Required)

The key conclusions are significant in that they advance our understanding of how cell cycle regulatory mechanisms can be adapted in cell types with short cell cycles. Results are also relevant in particular for the biology of T cells, and the finding that Emi1 controls the cell cycle and cell fate in CD8 T cells is noteworthy. These represent advances in our mechanistic understanding of cell division. In addition to the significance of the conclusions, the greatest strength of the manuscript is the technical quality of the proteomics data and the fair conclusions drawn from them. The results will be of interest to scientists studying basic research in cell, immune, and cancer biology.

The greatest limitation of the study is the lack of insights into how Emi1 is driving S phase and how its loss leads to accumulation of cells in G1. This result is quite interesting, because it is opposite what may be expected considering the known function of Emi1 as an APC/C substrate inhibitor and the observation in Fig. 5 that APC/C substrates are similarly low in CD8 T cells and 3T3 cells. This discrepancy and the logic of testing Emi KO should at least be more thoroughly discussed, and additional experiments, as described above, would increase the impact of the study. A second limitation is the lack of conclusions from many comparisons of protein levels among the cell type and cell-cycle phases. For example, the significance of similarities and differences in levels of the Cdks (Fig. 4) and Cdh1 (Fig. 5) is left undiscussed.

3. How much time do you estimate the authors will need to complete the suggested revisions:

Estimated time to Complete Revisions (Required)

(Decision Recommendation)

Between 1 and 3 months

4. *Review Commons* values the work of reviewers and encourages them to get credit for their work. Select 'Yes' below to register your reviewing activity at Web of Science Reviewer Recognition Service (formerly Publons); note that the content of your review will not be visible on Web of Science.

Web of Science Reviewer Recognition

Yes

Review #3

1. Evidence, reproducibility and clarity:

Evidence, reproducibility and clarity (Required)

Summary:

In this paper, the authors combine advanced quantitative mass spectrometry-based proteomics with the PRIMMUS cell sorting technique to profile cell cycle proteins across the G1, S, and G2/M phases in CD8 T cells, embryonic stem cells (mESCs), and NIH3T3 fibroblasts. Their well-designed experimental approach-avoiding artifacts from synchronization-yields an impressive and detailed dataset on cell cycle dynamics. The experimental approach to interrogate three different cell lines for cell cycle protein dynamics, without using synchronization, is technically excellent. The data extracted from that initial experiment, which is shown in figures 2, 3, 4 and 5, is clearly described and well presented.

My concern is with conceptual advance of study. The author set out to determine how cell cycle control mechanisms could give rise to rapid proliferation in CD8 t cells, but it is not clear they have addressed that question. The data do not clearly support a model in which mESCs or CD8 T cells exhibit dampened cell cycle protein periodicity compared to 3T3 fibroblasts, nor for a change in APC/C being a key driver. Notably, the depletion of Emi1 appears to most significantly impact later cell cycle stages, as has been reported previously by others, and thus it is unclear that in this system it is promoting S-phase entry. And, while the impact on re-replication is clear, this is a very well reported phenotype. Overall, I am very impressed by the technical aspects of the study, but thought the authors could put more effort toward developing more meaningful interpretations of the data, which felt under-developed.

Comments and recommendations:

- There does not appear to be any statistical comparative analysis accompanying the proteomics data. While the differences highlighted in the text are generally clear from the figures, including statistical comparisons would strengthen the analysis and provide greater confidence in the reported findings.
- It is unclear what the box and whiskers represent in these plots. By convention, box plots typically display the interquartile range, with the central line indicating the median and the whiskers representing the minimum and maximum values or a defined range. If the authors

are using a different convention, it should be clearly stated in the figure legends. Alternatively, I recommend using a different data representation to avoid potential confusion.

- Line 133 and figure 2B and D. It's unclear to me what 'common cell cycle regulated protein' are. The methods: "Fold changes were calculated and ANOVAs performed to identify significantly changing proteins in each cell line using the following filtering criteria: p < 0.05 and fold change > 2." does not fully clarify the analysis. For example, it's not clear what the fold changes are being compared to-is it a specific cell cycle phase, a baseline, or another reference point? Additionally, is the reported p-value corrected for multiple hypothesis testing? Overall, the methodology could benefit from further clarification.
- In Figure 2B, it is unclear how the data are normalized. Is it appropriate to compare values across rows, columns, or both? Additionally, the ordering of the CD8 T cell G2/M samples appears to be ascending from top to bottom-what is the rationale behind this ordering? Clarifying the normalization method and how to interpret comparisons within the figure would help improve its readability. As it is now, I am not clear on how this data supports the statement where it is called in the text "There was a core set of 121 proteins that were consistently cell cycle regulated across all three cell types" (lines 130-132). It would be interesting to know how these data compare the authors prior proteomics data on cell cycle proteins and to that of authors.
- In Figure 2C, are these the most frequent GO terms among the "cell cycle-regulated proteins"? It is unclear whether this represents a curated list of commonly known cell cycle-related terms or if these are the top GO terms identified through enrichment analysis. Clarifying this distinction would help readers better understand how these terms were selected and what the figure is intended to convey.
- The interpretation of differences in some APC/C components is not clear to me, particularly given the relationship to APC/C substrate expression. For example, ANAPC10 is down in CD8 cells, but the APC/C appears highly active given the high differential between substrate abundance between G1 and S/G2/M cells.
- I have some concerns regarding the Emi1 knockout experiments. First, the knockout does not appear to be particularly efficient. In addition, the results do not necessarily support the proposed conclusion. If Emi1 was a key driver of G1/S progression, I would think you would observe more 2N cells, not fewer. The relationship to DNA damage, made in Fig 6f is unclear.
- In the flow cytometry data in Figure 6C, the Emi1 knockout cells are EdU-positive and have >4N DNA content. This is consistent with a re-replication phenotype and for a vital role of Emi1 in preventing re-activation of the APC/C in late S or G2 phase. It is difficult to square this with the assertion that it is promoting G1/S progression. Notably, this is a well reported Emi1 phenotype.

Minor comments:

- Some figures lack error bars (e.g., Figures 2C, 2D, and 5A). I also recommend including individual data points on all graphs, as this is now required by many journals. Since most of the graphs likely represent experimental triplicates, adding these elements should be straightforward and will allow readers to see actual spread of the data.
- The terminology used to refer to CD8 T cells is inconsistent across the manuscript-for example, "CD8T" in Figure 2 and "CD8" in Figure 3. Standardizing this terminology throughout the figures and text would improve clarity.
- The authors frequently refer to "increases in pRB-," which is somewhat confusing. I recommend rephrasing this as "decrease in pRB" to make the interpretation clearer and easier to follow.

2. Significance:

Significance (Required)

Significance and Advance

Strength: Excellent deep, quantitative proteomic dataset of cell cycle proteome dynamics across three independent cell lines. Done without chemical or other synchronization. This incrementally improves on previous proteomics datasets.

Weaknesses: Limited conceptual advance in our understanding of cell cycle proteomes, or insight into how different cell types proliferate at different rates.

Audience: interested audiences include those study cell cycle control, proteomics.

My expertise: cell cycle control and protein dynamics.

3. How much time do you estimate the authors will need to complete the suggested revisions:

Estimated time to Complete Revisions (Required)

(Decision Recommendation)

Between 1 and 3 months

4. *Review Commons* values the work of reviewers and encourages them to get credit for their work. Select 'Yes' below to register your reviewing activity at Web of Science Reviewer Recognition Service (formerly Publons); note that the content of your review will not be visible on Web of Science.

Web of Science Reviewer Recognition

No

Full Revision

Manuscript number: RC-2025-02944
Corresponding author(s): Tony Ly

[Please use this template only if the submitted manuscript should be considered by the affiliate journal as a full revision in response to the points raised by the reviewers.

If you wish to submit a preliminary revision with a revision plan, please use our "Revision Plan" template. ***It is important to use the appropriate template to clearly inform the editors of your intentions.****]*

1. General Statements [optional]

2. Point-by-point description of the revisions

This section is mandatory. Please insert a point-by-point reply describing the revisions that were already carried out and included in the transferred manuscript.

Reviewer #1 (Evidence, reproducibility and clarity (Required)):

In this study by Lewis and colleagues, the authors use highly quantitative proteomics to compare cell cycle patterns in T lymphocytes to mouse embryonic stem cells and differentiated fibroblasts. The work is significant because the cell cycle field has been dominated by only a few paradigms based on model systems such as yeasts, cancer-derived cell lines, and fibroblasts. The different cell cycles in other cell types have not been thoroughly investigated because they are less experimentally tractable. Prior studies have drawn conclusions about embryonic stem cells that turned out later to be primarily from attempts to induce synchronization prior to analysis. Here, the authors apply their previously-described method for detecting proteins according to cell cycle phase by flow-sorting and then mass spectrometry. They find expected cell cycle fluctuations in the three cell lines, but also a few unexpected differences. Primarily, cyclin E protein appears to be expressed at higher levels in the fast-proliferating mESCs and CD8 cells compared to NIH3TS and to be less cell cycle-regulated. The mechanism and functional relevance for this difference are not considered.
The authors chose to focus several experiments on the APC/C ubiquitin ligase inhibitor, Emi1, which they find appropriately cell cycle-regulated in CD8 and mESCs but expressed at higher levels in CD8 cells. They show that CD8 differentiation is associated with cell cycle changes and that perturbing the cell cycle in CD8 cells affects their differentiation (which is not surprising). Altogether this study provides an excellent resource for the field from the detailed high-quality proteomics data sets (though see point #8). On the other hand, the experiments with EMI1-KO and differentiating cells seem largely unrelated to the cell cycle analysis itself; these results may be mostly indirect consequences of cell cycle perturbation rather than specific to EMI1 function.

Full Revision

This is a very thorough, rigorous, and valuable proteomics characterization of cell cycle differences, but the choice of follow-up functional experiments isn't logical in my opinion.

Major points:
1. It is not clear how the quantification accounts for differences in cell size or nuclear/cytoplasmic fractions among the different cell types. For example, ESCs are typically small with relatively less cytoplasm. Since a great many cell cycle proteins are nuclear, normalizing to total protein analyzed and not cell number could make these proteins appear more abundant. In other words, does 1 ug of protein from mESCs represent the same number of cells or nuclei as 1 ug of protein from NIH3T3?

We have used the proteomic ruler[1] approach to estimate the protein content of subcellular compartments between cell types, as has been done by my lab[2,3] and others[4]. We have limited this analysis to a single cell cycle phase, namely G2&M. As shown, CD8 T cells have a higher nuclear fraction than other cells. However, the difference between mESC and NIH3T3 is minor.

Figure for referee with unpublished data not shown.

We have calculated the amount of protein per cell also using the proteomic ruler. This shows the protein content of G2&M phase cells varies with cell type. Therefore, proportionally, 1 µg of protein represents fewer mESC and NIH3T3 cells compared to CD8 T cells. Notably, our observation that cell cycle regulated proteins are generally higher abundance in faster dividing cells (mESC and CD8 T cells) does not follow the same trend. Therefore, it is unlikely that the observations we made are due to normalization.

Figure for referee with unpublished data not shown.

The proteomic ruler normalizes MS intensities for individual proteins by histone intensities. While the cytoplasmic fraction may vary, the diploid murine cell types used in this study have the same genome size and similar histone content per cell. Thus, this normalization produces an estimate of protein copies per cell. Notably, values normalized this way still support the assertion that cell cycle regulated proteins are present at higher abundance in mESCs and CD8 T cells.

For illustration, we have shown parts-per-million (ppm) and protein copies side-by-side here for Cyclin A2.

Figure for referee with unpublished data not shown.

These data show that Cyclin A2 is present in higher abundance in mESCs and CD8s. Considering that the difference in total protein content between cell type is much greater than differences in nuclear protein content, we would argue that comparing the effective concentration (ppm) is more appropriate than protein copies.

2. The motivation for the project was given as wanting to understand if fast-cycling CD8 T cells resemble fast-cycling mESCs, which the authors show they partially do. The molecular phenotype in common is cyclin E abundance, but we are left without an understanding for how that occurs. Are there differences in expression of the E3 ligase for cyclin E?

Thanks to feedback from another review, we have re-evaluated our analysis of APC/C substrates, using an expanded list of APC/C substrates curated by the Davey group (ICR, London). We now evaluate 43 APC/C substrates (compared to 26 in the previous version of the manuscript), that have also been selected for increased abundance in G2&M phase compared to G1 phase, in at least one cell type, as would be expected for a bona fide substrate. This showed that in G1, APC/C substrate levels were higher in both mESC and CD8 T cells, compared to NIH3T3. In evaluating the multiple, potentially exciting avenues to explore, we also considered the technical feasibility and likelihood of success of perturbation experiments with Cyclin E versus Emi1, since Cyclin E function is shared between two paralogues. Indeed, Cyclin E1 nullizygous mice show no overt phenotypes[5], whereas Emi1 deletion is embryonic lethal[6]. We have added text to expand the rationale.

However, we take the reviewer's point that more can be done to address the regulation of Cyclin E1. Therefore, the revised manuscript includes a new Figure 5 that investigates this further. We

show that Cyclin E1 degradation is proteasome and Neddylation dependent, suggesting that Cyclin E1 is targeted for degradation by a cullin ring ligase (CRL) in CD8 T cells and NIH3T3 cells. Cyclin E1 protein has a similar half-life in CD8 T cells and NIH3T3 cells. These results suggest that higher Cyclin E1 levels may be due to higher transcription. As described in the Discussion, further work will be required to elucidate the mechanisms and are outside the scope of the present manuscript

3. We also don't learn if the constitutive cyclin E is why cells cycle quickly because no experiments to change cyclin E are provided.

We now include additional data (Fig. 5) showing that overexpression of Cyclin E1 in NIH3T3 increased S-phase frequencies but reduced cell numbers. These results suggest that Cyclin E1 promotes S-phase entry in CD8 T cells, but additional mechanisms may be present to prevent replication stress-induced arrest.

4. In an unexpected turn for the project, the authors generated inducibly Emi1-null CD8 T cells and analyzed them for replication, re-replication, and phosphorylated Rb. This section is fine, but the rationale isn't obvious, particularly if constitutive cyclin E (not an APC/C substrate) is the proposed driver of fast proliferation. Emi1 isn't the general reason for fast proliferation if it is only high during G1 for CD8 and not mESCs. Emi1 loss has also been known to promote re-replication for quite a few years, a finding the authors cite. The replication-dependent DNA damage and cell cycle arrest are both well-known outcomes from re-replication. So, although the data are of good quality, I'm not sure that showing Emi1 has the same role in CD8 cells as what was reported in other cells adds much to the field. The authors could choose to save Figure 6 for a more APC/C-focused study and use EMI1 as a general cell proliferation marker in Figure 7.

We now show additional data explaining the rationale for follow up experiments on Emi1 (Fig. 6, Fig. 7b-c). We note that cell cycle arrest in early G1 is consistent with a role of Emi1 in promoting G1 progression. Based on the lack of gammaH2A.x signal in early G1 Emi1KO cells, we would argue that this arrest is unlikely due to re-replication.

5. What is the cell cycle distribution of the IL-15 and IL-2 differentiated cells? If they are different, then some of the protein abundance changes to regulated proteins such as p21 might be a symptom of cell cycle differences rather than differentiation. It seems IL-15 is more cell cycle promoting form Figure S3, but that isn't clear from the assays presented.

We now show that cell cycle distributions for IL15 and IL2 treated CD8 T cells in Fig. S4a and here for convenience. Their cell cycle profiles are comparable.

Figure for referee with unpublished data not shown.

6. The change in the CD62L marker is argued to be unrelated to cell cycle and somehow more directly downstream of Emi1. The connection here is tenuous, in part because there's no comparison to a similar cell cycle perturbation independent of Emi1. How would Emi1 modulate CD62L if CDC62L does not show evidence of being an APC/C substrate?

We have removed the data on CD62L as we thought it distracted from the main focus of the manuscript, which is to study cell cycle control mechanisms.

7. As presented, Emi1 has a unique effect on the immune phenotype. It is also possible that many cell cycle perturbations would similarly affect differentiation and not only Emi1. Can the authors distinguish Emi1-specific effects from more common cell cycle perturbation effects? Alternatively, interpreting the EMI1-KO data more generally could avoid confusion.

See above.

8. The authors should provide legends to the supplementary tables in the text and/or the tables themselves. Several column headers in the tables are not defined.

We have added legends to the supplementary tables.

Minor points:
a. Figure 4g is titled Cdkn2a (which would be p16) but the legend says its Cdkn1a (p21). The p21 protein was undetected in NIH3T3? One would expect it to be in the G1 cells at least.

The legend should have stated p16. This is now corrected. p21 was not detected in this dataset.

b. Line 56: cyclin E is not an APC/C substrate as implied by the text. If Skp2 is a bona fide substrate (not sure that's true), then APC/C activity would have opposite effects on Cyclin E vs Cyclin A.

We did not intend to imply this and apologize for the confusing sentence. The sentence now reads, “Mechanistically, the levels of Cyclin A and additional cell cycle promoting proteins are sustained in mESCs by inhibition of the key E3 ligase, the APC/C, by its protein inhibitor, Emi1 (Fbxo5)”.

c. What is the red arrow in Figure 6C?

The red arrows indicate re-replicating cells. We have added this clarification to the figure legend.

d. Figure 7b is protein copy number whereas all other graphs are for ppm. The legend on line 686 has "cite immPRES" but this citation didn't make it into the submitted manuscript.

This is now corrected.

Reviewer #1 (Significance (Required)):

The work is significant because the cell cycle field has been dominated by only a few paradigms based on model systems such as yeasts, cancer-derived cell lines, and fibroblasts. The different cell cycles in other cell types have not been thoroughly investigated because they are less experimentally tractable. Prior studies have drawn conclusions about embryonic stem cells that turned out later to be primarily from attempts to induce synchronization prior to analysis. This is a very thorough, rigorous, and valuable proteomics characterization of cell cycle differences, but the choice of follow-up functional experiments isn't logical in my opinion.

Reviewer #2 (Evidence, reproducibility and clarity (Required)):

Summary
This study uses proteomics as an approach to understand regulatory mechanisms contributing to a short cell division cycle in CD8 T cells. While the cell cycle machinery has been studied in mouse embryonic stem cells (mESCs) and these discoveries have provided a framework for understanding atypically short cell cycles, whether those mechanisms are conserved in other cell types that undergo a short cycle has not been clear. Analysis of the cellular proteome was performed at three different cell-cycle stages (G1, S, G2/M) and across three cell lines (CD8 T cells and mESCs and fibroblast 3T3 cells as comparators for short and normal cycles, respectively). An important innovation here is the application of a PRIMMUS protocol, which uses markers of cell cycle stage and sorting rather than treatments, such as inhibitors, that can have unwanted perturbations. The authors find both similarities and differences between CD8 T cells and mESCs. Of particular interest, CycE levels are constitutively high in both cell types with a short cycle compared to fibroblast cells, but APC/C substrates are uniquely lower during

G1 in CD8 T cells. Using CRISPR knock-out, the APC/C inhibitor Emi1 is then implicated as a key regulator of G1/S control in CD8 T cells as well as phenotypic fate.

Major Comments:

Overall, the conclusions are fairly made from the data. In particular, the proteomics data and analysis are of high technical quality. The data and methods are clearly presented in a manner that could be reproduced, and experiments are, with one exception noted gere, performed with appropriate replicates and statistical analysis. I have only two major comments.

1) From the description of the CRISPR methods and results, the origin of the Emi 1KO cells used in all the experiments is unclear. Only one Emi1 KO sample or experiment is shown along with control in each panel. A number of questions should be answered in order to clarify.

We now include new data and clarifications to answer the reviewer's questions in the manuscript and below.

Was this sample originated from the total cells grown out of the gRNA treatment (and on which the TIDE analysis was performed)?

Samples for flow cytometry, TIDE and western blotting analysis were cells grown out of the gRNA treatment.

If so, which exon was the gRNA targeting in the cells used in subsequent experiments?

Were single clones ever grown? Ideally, experiments should be performed with multiple single clonal lines, and at least one from each exon used, if possible. Alternatively, a population generated from each of the two guids should be used in all the experiments as a proper biological replicate.

Clonal selection is not possible because these are ex vivo primary cell cultures that cannot be cultured indefinitely. Furthermore, the deleted gene is intracellular and in the absence of a reporter, it is impossible to FACS select $Emi1^{KO}$ cells.

Preliminary experiments used each gRNA separately. These experiments are now shown in Fig. S2b-d. All subsequent experiments were performed with pooled gRNA as this resulted in a more penetrant phenotype, as shown in Fig. 7d. Notably, the percentage of re-replicating cells is higher using pooled gRNAs (c.f. Fig. 7d and Fig. S3d), and a typical, asynchronous cell cycle profile is largely restored two days following KO using each guide separately (Fig. S3d), whereas a cell cycle defect is still observable at this timepoint using the pooled gRNA approach (Fig. 7d).

The Results and Discussion sections have been amended to clarify.

It may be that homozygous deletion of Emi1 is not possible because of the effects on proliferation. If so, the authors should comment on this along with why the protein level in Sup. Fig. 2b is still so high in the KO line. Considering the inefficiency of the overall KO, it is especially important to understand Emi1 expression throughout the polyclonal population to interpret the results. Are there some cells expressing normal levels and some cells expressing no Emi1? If monoclonal, are the observed effects from haploinsufficiency? More description of the method and caveats to the interpretation of results are needed here, and results would be made more robust if more than one clone or gRNA treatment were used. It would be expected this may take 2-3 months with reasonable resources to perform this additional analysis, if not done already.

As mentioned above, we now include data from independent gRNAs.

We also have amended the text to explain how the data should be interpreted in the context of an incomplete knock out.

We do not have evidence that Emi1 is haploinsufficient. $Emi1^{+/-}$ animals are viable and display no overt phenotypes compared to $Emi1^{+/+}$ (Lee et al. *Mol Cell Biol* 2006, https://pmc.ncbi.nlm.nih.gov/articles/PMC1592703/).

2) OPTIONAL. One shortcoming of the manuscript is the lack of insights into why knock out of Emi1 leads to G1 arrest. Follow up experiments on the APC/C substrates in Fig. S1b or analysis of proteins that are upregulated or downregulated by Emi KO would increase the significance of the manuscript. These experiments may be time consuming and could be considered beyond the scope of this study, but they would increase the impact of the study.

This is an excellent suggestion. We will pursue this in future studies.

Minor comments:
1) Some brief aspects of the methods could be added to the description of results to help improve clarity in a few places. For example, the threshold for including the 121 proteins in Fig. 2b and what antibodies were used for staining in the PRIMMUS approach would be important enough that it should be stated in results with no need to look up detailed method.

These details have now been added. We have added method details throughout the manuscript.

2) It should be clarified that in most cases, use of the term "abundance" in comparing protein levels is actually a relative abundance for a specific cell type. For example in the following sentence, relative and not absolute abundances are being compared : "In mESC, there was higher abundance in G1 phase compared to CD8 T cells and NIH3T3 (Fig.136 2d)." Clarifying

upon first use of the term abundance would be sufficient.

We have added a sentence to clarify.

3) In some of the protein abundance panels there does not appear to be any mark in the graph, which is consistent with there being no observation of protein. This absence of any observation should be made explicit in the figure captions.

We have added this to the figure legends.

4) It should be clarified with its first use (line 264) that "pRb" means phosphorylated Rb.

This is now defined earlier.

Reviewer #2 (Significance (Required)):

The key conclusions are significant in that they advance our understanding of how cell cycle regulatory mechanisms can be adapted in cell types with short cell cycles. Results are also relevant in particular for the biology of T cells, and the finding that Emi1 controls the cell cycle and cell fate in CD8 T cells is noteworthy. These represent advances in our mechanistic understanding of cell division. In addition to the significance of the conclusions, the greatest strength of the manuscript is the technical quality of the proteomics data and the fair conclusions drawn from them. The results will be of interest to scientists studying basic research in cell, immune, and cancer biology.

The greatest limitation of the study is the lack of insights into how Emi1 is driving S phase and how its loss leads to accumulation of cells in G1. This result is quite interesting, because it is opposite what may be expected considering the known function of Emi1 as an APC/C substrate inhibitor and the observation in Fig. 5 that APC/C substrates are similarly low in CD8 T cells and 3T3 cells. This discrepancy and the logic of testing Emi KO should at least be more thoroughly discussed, and additional experiments, as described above, would increase the impact of the study. A second limitation is the lack of conclusions from many comparisons of protein levels among the cell type and cell-cycle phases. For example, the significance of similarities and differences in levels of the Cdks (Fig. 4) and Cdh1 (Fig. 5) is left undiscussed.

Reviewer #3 (Evidence, reproducibility and clarity (Required)):

Summary:

In this paper, the authors combine advanced quantitative mass spectrometry-based proteomics with the PRIMMUS cell sorting technique to profile cell cycle proteins across the G1, S, and G2/M phases in CD8 T cells, embryonic stem cells (mESCs), and NIH3T3 fibroblasts. Their well-designed experimental approach-avoiding artifacts from synchronization-yields an impressive and detailed dataset on cell cycle dynamics. The experimental approach to interrogate three different cell lines for cell cycle protein dynamics, without using synchronization, is technically excellent. The data extracted from that initial experiment, which is shown in figures 2, 3, 4 and 5, is clearly described and well presented.
My concern is with conceptual advance of study. The author set out to determine how cell cycle control mechanisms could give rise to rapid proliferation in CD8 t cells, but it is not clear they have addressed that question. The data do not clearly support a model in which mESCs or CD8 T cells exhibit dampened cell cycle protein periodicity compared to 3T3 fibroblasts, nor for a change in APC/C being a key driver. Notably, the depletion of Emi1 appears to most significantly impact later cell cycle stages, as has been reported previously by others, and thus it is unclear that in this system it is promoting S-phase entry. And, while the impact on re-replication is clear, this is a very well reported phenotype. Overall, I am very impressed by the technical aspects of the study, but thought the authors could put more effort toward developing more meaningful interpretations of the data, which felt under-developed.

We respectfully disagree. Our study provides an important conceptual advance by correcting existing models established in the field. For example, in an excellent review in 2020, dampened APC/C activity and Cyclin A2 fluctuations were proposed to promote rapid cell cycles based on literature dating to early 2000s using techniques that were at the state-of-the-art of that time. Using current state-of-the-art approaches, we now revisit the question, which show quantitative data that do not support these models.

We agree that additional work will be required to understand what promotes rapid cell divisions. Thanks to feedback from the reviewers, we have re-evaluated our analysis of APC/C substrates, using an expanded list of APC/C substrates curated by the Davey group (ICR, London). We now evaluate 43 APC/C substrates (compared to 26 in the previous version of the manuscript), that have also been selected for increased abundance in G2&M phase compared to G1 phase, in at least one cell type, as would be expected for a bona fide substrate. This showed that in G1, APC/C substrate levels were higher in both mESC and CD8 T cells, compared to NIH3T3. In evaluating the multiple, potentially exciting avenues to explore, we also considered the technical feasibility and likelihood of success of perturbation experiments with Cyclin E versus Emi1, since Cyclin E function is shared between two paralogues. Indeed, Cyclin E1 nullizygous mice show no overt phenotypes[5], whereas Emi1 deletion is embryonic lethal[6]. We have added text to expand the rationale.

Our data suggest that Emi1 plays a role (e.g. Fig. 7d-g), but that Emi1 likely has a more important role in safeguarding against re-replication. As Reviewer 1 points out, an interesting

and promising avenue of inquiry is the constitutive expression of Cyclin E1 in both mESC and CD8 T cells.

To further address this, the revised manuscript includes a new Figure 5. We show that Cyclin E1 degradation is proteasome and Neddylation dependent, suggesting that Cyclin E1 is targeted for degradation by a cullin ring ligase (CRL) in CD8 T cells and NIH3T3 cells. Additionally, Cyclin E1 protein has a similar half-life in CD8 T cells and NIH3T3 cells. These results suggest that Cyclin E1 levels may be transcriptionally controlled. As described in the Discussion, further work will be required to elucidate the mechanisms and are outside the scope of the present manuscript. We also include additional data showing that overexpression of Cyclin E1 increased S-phase frequency in NIH3T3 cells, but reduced cell numbers. This is consistent with previous work showing that high Cyclin E1 levels can induce replication stress (Diffley and Cook labs). Interestingly, together these results suggest that while Cyclin E1 can promote S-phase entry in CD8 T cells, additional mechanisms are likely to prevent replication stress-induced cell cycle arrest.

Comments and recommendations:
• There does not appear to be any statistical comparative analysis accompanying the proteomics data. While the differences highlighted in the text are generally clear from the figures, including statistical comparisons would strengthen the analysis and provide greater confidence in the reported findings.

Due to the high number of contrasts and quantitative figures shown, we do not think it would be clearer to add contrast bars to the graphs and detract from the data, which we agree are generally clear from the figures already. Results of statistical tests are reported in Supplementary Table 2. We have also changed graphs to display individual points, as the reviewer suggested, and added p-values to the text where appropriate.

• It is unclear what the box and whiskers represent in these plots. By convention, box plots typically display the interquartile range, with the central line indicating the median and the whiskers representing the minimum and maximum values or a defined range. If the authors are using a different convention, it should be clearly stated in the figure legends. Alternatively, I recommend using a different data representation to avoid potential confusion.

These have been changed to bar plots with error bars displaying standard error of the mean. Figure legends have been updated.

• Line 133 and figure 2B and D. It's unclear to me what 'common cell cycle regulated protein' are. The methods: "Fold changes were calculated and ANOVAs performed to identify significantly changing proteins in each cell line using the following filtering criteria: $p < 0.05$ and fold change > 2." does not fully clarify the analysis. For example, it's not clear what the fold changes are being compared to-is it a specific cell cycle phase, a baseline, or another reference

point? Additionally, is the reported p-value corrected for multiple hypothesis testing? Overall, the methodology could benefit from further clarification.

We have now amended the text to the following, which we think is clearer, “ANOVA and Tukey’s multiple comparison test were performed to test for significant differences in protein abundance between cell cycle phases. Fold changes were calculated for all pairwise contrast. This statistical analysis was repeated separately for each cell type. Proteins were then deemed significantly changing if they met the following filtering criteria: $p < 0.05$ and fold change > 2. The p values are unadjusted for multiple hypothesis testing.”

• In Figure 2B, it is unclear how the data are normalized. Is it appropriate to compare values across rows, columns, or both? Additionally, the ordering of the CD8 T cell G2/M samples appears to be ascending from top to bottom-what is the rationale behind this ordering? Clarifying the normalization method and how to interpret comparisons within the figure would help improve its readability. As it is now, I am not clear on how this data supports the statement where it is called in the text "There was a core set of 121 proteins that were consistently cell cycle regulated across all three cell types" (lines 130-132). It would be interesting to know how these data compare the authors prior proteomics data on cell cycle proteins and to that of authors.

The heatmap shows row-scaled protein abundances. This is now clarified in the Figure legend. We have amended the heatmap to include a scatter plot showing the average scaled protein abundances for each cell type/cell cycle phase. We think this graph better illustrates the point we are making.

We agree a meta-analysis of proteomics datasets characterizing cell cycle control would be of interest. Indeed, initially we carried out comparisons between our prior work in NB4 and CD8 T cells. However, most of this type of work, including from our lab and others, has been carried out in human cell lines. Comparison between human and rodent cell lines is not trivial because there have been reports that some proteins are cell cycle regulated in one organism, but not the other, e.g. Orc1[9,10]. This motivated us to compare only mouse cell types to avoid species-specific differences in regulation. We have added this rationale to the Results section.

Moreover, technical differences in synchrony and MS analysis approach would limit the interpretation. Differences in synchrony would be especially problematic since we are specifically evaluating the magnitude of cell cycle regulated protein changes. We agree however that such an analysis would be of interest, and something to pursue systematically in the future, but is beyond the scope of the current study.

• In Figure 2C, are these the most frequent GO terms among the "cell cycle-regulated proteins"? It is unclear whether this represents a curated list of commonly known cell cycle-related terms or if these are the top GO terms identified through enrichment analysis. Clarifying this distinction

would help readers better understand how these terms were selected and what the figure is intended to convey.

This was a GO term enrichment analysis, as explained in the Figure legend. We have clarified this in the main text.

• The interpretation of differences in some APC/C components is not clear to me, particularly given the relationship to APC/C substrate expression. For example, ANAPC10 is down in CD8 cells, but the APC/C appears highly active given the high differential between substrate abundance between G1 and S/G2/M cells.

We have removed this section of the Results as we feel it is a distraction from the flow of the experiments.

• I have some concerns regarding the Emi1 knockout experiments. First, the knockout does not appear to be particularly efficient.

Our approach is straightforward compared to the generation of a complete, conditional knockout of Emi1 in primary ex vivo murine CD8+ T cells. This would be technically demanding, costly, and require several years of experimental work. Genetic manipulation of these cells is limited by the short time period (~1 week) in which these cells can be cultured, before cells require restimulation. Notably, restimulation of cells exposed to antigen produces CD8 T cells that are phenotypically and immunologically distinct from the activation of naïve T cells.

We agree that in vitro delivery of Cas9-gRNA has limitations. Electroporation is generally efficient, as shown by treatment of CD8 T cells with Cas9-gRNA targeting the fitness-neutral cell surface receptor Thy1 (see below). However, indel efficiencies vary with locus, and disruption of genes required for proliferation promote outgrowth of cells with the intact locus.

Figure for referee with unpublished data not shown.

Outgrowth is particularly acute for CD8 T cells. CD8 T cells can undergo ~4 doublings over a 24-hour period. Thus, even in the hypothetical case of 95% efficiency, the remaining 5% increases to 46% by the 24-hour harvest timepoint.

Review COMMONS

We note that the % indel frequencies and % reduction in Emi1 protein by immunoblot (~50%), correlate with re-replication, the major phenotype of Emi1 deletion.

In the revised manuscript, we have renamed these cells CD8 T + Cas9-gEmi1$^{(ex2+ex4)}$, which reflects the experimental treatment and less likely to prompt confusion about the nature of the resulting cells. We have also changed the Results and Discussion sections to clarify the experiments and the limitations of the approach.

In addition, the results do not necessarily support the proposed conclusion. If Emi1 was a key driver of G1/S progression, I would think you would observe more 2N cells, not fewer. The relationship to DNA damage, made in Fig 6f is unclear.

The decrease in 2N is due to a substantial increase in >4N cells. To avoid this confound, we specifically examined 2N cells using Rb phosphorylation as a marker of G0/early G1 (phospho-Rb negative) and late G1 (phospho-Rb positive). As shown in Fig. 7f, the proportion of G0/early G1 cells is increased, suggesting that loss of Emi1 results in G0/early G1 arrest. The increased proportion of G0/early G1 cells is not due to DNA damage, as evidenced by gamma H2A.x staining (Fig. 7g). We agree that caution is needed in interpreting kinetics from a fixed cell assay. Further work, for example, using live cell imaging, would be important to further show that cells arrest in G0/G1. We have included additional text in the Discussion to clarify this point.

• In the flow cytometry data in Figure 6C, the Emi1 knockout cells are EdU-positive and have >4N DNA content. This is consistent with a re-replication phenotype and for a vital role of Emi1 in preventing re-activation of the APC/C in late S or G2 phase. It is difficult to square this with the assertion that it is promoting G1/S progression. Notably, this is a well reported Emi1 phenotype.

We agree that a major phenotype following Emi1 deletion is re-replication. Indeed, the main text describes this and cites original work in human cancer cell lines first reporting this function. Our data also suggest a function of Emi1 in G1 progression, as described above. We note that it is not uncommon for one protein to have multiple functions, particularly in the context of the cell cycle, in which protein function can be cell cycle dependent. We agree however that the major function of Emi1 appears to be safeguarding against re-replication, and that it has an additional function in G1 progression.

Minor comments:

• Some figures lack error bars (e.g., Figures 2C, 2D, and 5A). I also recommend including individual data points on all graphs, as this is now required by many journals. Since most of the graphs likely represent experimental triplicates, adding these elements should be straightforward and will allow readers to see actual spread of the data.

Individual data points have been added to graphs.

• The terminology used to refer to CD8 T cells is inconsistent across the manuscript-for example, "CD8T" in Figure 2 and "CD8" in Figure 3. Standardizing this terminology throughout the figures and text would improve clarity.

To clarify, we now use “CD8 T cells” in the main text and figure legends, and “CD8” in figure labels.

• The authors frequently refer to "increases in pRB-," which is somewhat confusing. I recommend rephrasing this as "decrease in pRB" to make the interpretation clearer and easier to follow.

We believe there might be a misunderstanding here and apologise for the lack of clarity. What we measure is the frequency of cells that have low or no phosphorylated Rb at S807/S811. In most instances in the text, we have made this as clear as possible by referring to the “frequency of G1pRb- cells”. However, there is one instance where the text is not quite clear, e.g. “However, $G1^{pRb-}$ cells showed no difference in gammaH2A.x staining between control and Emi1KO cells. This suggests that the increased frequency of $G1^{pRb-}$ and decreased S-phase frequencies…“. This is now changed to “However, $G1^{pRb-}$ cells showed no difference in gammaH2A.x staining between control and Emi1KO cells. This suggests that the increased frequency of $G1^{pRb-}$ cells and decreased S-phase frequency…”.

Reviewer #3 (Significance (Required)):

Significance and Advance
Strength: Excellent deep, quantitative proteomic dataset of cell cycle proteome dynamics across three independent cell lines. Done without chemical or other synchronization. This incrementally improves on previous proteomics datasets.
Weaknesses: Limited conceptual advance in our understanding of cell cycle proteomes, or insight into how different cell types proliferate at different rates.
Audience: interested audiences include those study cell cycle control, proteomics.
My expertise: cell cycle control and protein dynamics.

References

1. Wiśniewski, J. R., Hein, M. Y., Cox, J. & Mann, M. A “Proteomic Ruler” for Protein Copy Number and Concentration Estimation without Spike-in Standards*. *Molecular & Cellular Proteomics* **13**, 3497–3506 (2014).
2. Crozier, L. *et al.* CDK4/6 inhibitor-mediated cell overgrowth triggers osmotic and replication stress to promote senescence. *Mol Cell* **83**, 4062-4077.e5 (2023).
3. Foy, R. *et al.* Oncogenic signals prime cancer cells for toxic cell overgrowth during a G1 cell cycle arrest. *Mol Cell* **83**, 4047-4061.e6 (2023).

4. Howden, A. J. M. *et al.* Quantitative analysis of T cell proteomes and environmental sensors during T cell differentiation. *Nat Immunol* **20**, 1542–1554 (2019).
5. Geng, Y. *et al.* Cyclin E Ablation in the Mouse. *Cell* **114**, 431–443 (2003).
6. Lee, H. *et al.* Mouse emi1Has an Essential Function in Mitotic Progression during Early Embryogenesis. *Mol Cell Biol* **26**, 5373–5381 (2006).
7. Lelliott, E. J. *et al.* CDK4/6 inhibition promotes antitumor immunity through the induction of T-cell memory. *Cancer Discov* **11**, 2582–2601 (2021).
8. Heckler, M. *et al.* Inhibition of cdk4/6 promotes cd8 t-cell memory formation. *Cancer Discov* **11**, 2564–2581 (2021).
9. Méndez, J. *et al.* Human Origin Recognition Complex Large Subunit Is Degraded by Ubiquitin-Mediated Proteolysis after Initiation of DNA Replication. *Mol Cell* **9**, 481–491 (2002).
10. McNairn, A. J., Okuno, Y., Misteli, T. & Gilbert, D. M. Chinese hamster ORC subunits dynamically associate with chromatin throughout the cell-cycle. *Exp Cell Res* **308**, 345–356 (2005).

Dear Dr. Ly,

Thank you for the transfer of your revised manuscript from Review Commons to EMBO reports. I have now received the reports from the three referees that I asked to re-evaluate the study, you will find below.

As you will see, the referees fully support the publication of the study in EMBO reports.

The manuscript now needs formatting according to our journal style, I ask you to do in a further revised manuscript. Please carefully review the instructions that follow below.

PLEASE NOTE THAT upon resubmission revised manuscripts are subjected to an initial quality control prior to exposition to re-review. Upon failure in the initial quality control, the manuscripts are sent back to the authors, which may lead to delays. Frequent reasons for such a failure are the lack of the data availability section (please see below) and the presence of statistics based on n=2 (the authors are then asked to present scatter plots or provide more data points).

When submitting your revised manuscript, we will require:

1) a .docx formatted version of the final manuscript text (including legends for main figures, EV figures and tables), but without the figures included. Figure legends should be compiled at the end of the manuscript text.

2) individual production quality figure files as .eps, .tif, .jpg (one file per figure), of main figures and EV figures. Please upload these as separate, individual files upon re-submission.

The Expanded View format, which will be displayed in the main HTML of the paper in a collapsible format, has replaced the Supplementary information. You can submit up to 6 images as Expanded View. Please follow the nomenclature Figure EV1, Figure EV2 etc. The figure legend for these should be included in the main manuscript document file in a section called Expanded View Figure Legends after the main Figure Legends section. Additional Supplementary material should be supplied as a single pdf file labeled Appendix. The Appendix should have page numbers and needs to include a table of content on the first page (with page numbers) and legends for all content. Please follow the nomenclature Appendix Figure Sx, Appendix Table Sx etc. throughout the text, and also label the figures and tables according to this nomenclature.

For more details, please refer to our guide to authors:
https://link.springer.com/journal/44319/submission-guidelines#cms-Revised-submissions

Please consult our guide for figure preparation:
https://media.springernature.com/original/springer-cms/rest/v1/content/27825798/data/v1

3) a complete author checklist, which you can download from our author guidelines (https://link.springer.com/journal/44319/submission-guidelines#cms-Revised-submissions). Please insert page numbers in the checklist to indicate where the requested information can be found in the manuscript. The completed author checklist will also be part of the RPF.

Please also follow our guidelines for the use of living organisms, and the respective reporting guidelines:
https://link.springer.com/partners/embo-press/editorial-policies#Animal%20and%20human%20studies

4) that primary datasets produced in this study (e.g. RNA-seq, ChIP-seq, structural and array data) are deposited in an appropriate public database. If no primary datasets have been deposited, please also state this in a dedicated section (e.g. 'No primary datasets have been generated and deposited'), see below.

See also: https://link.springer.com/partners/embo-press/editorial-policies#Data%20deposition

Please remember to provide a reviewer password if the datasets are not yet public.

The accession numbers and database should be listed in a formal "Data Availability" section that follows the model below. This is now mandatory (like the COI statement). Please note that the Data Availability Section is restricted to new primary data that are part of this study. This section is mandatory. As indicated above, if no primary datasets have been deposited, please state this in this section

Data availability

The datasets produced in this study are available in the following databases:

- RNA-Seq data: Gene Expression Omnibus GSE46843 (https://www.ncbi.nlm.nih.gov/geo/query/acc.cgi?acc=GSE46843)
- [data type]: [name of the resource] [accession number/identifier/doi] ([URL or identifiers.org/DATABASE:ACCESSION])

*** Note - All links should resolve to a page where the data can be accessed. ***

See also: https://link.springer.com/partners/embo-press/editorial-policies#Data%20availability%20statement

Moreover, I have these editorial requests:

5) We now request the publication of original source data with the aim of making primary data more accessible and transparent to the reader. You will receive a separate email with instructions for providing source data with your revised manuscript, including information how to upload and organize the files.

6) Our journal encourages inclusion of *data citations in the reference list* to directly cite datasets that were re-used and obtained from public databases. Data citations in the article text are distinct from normal bibliographical citations and should directly link to the database records from which the data can be accessed. In the main text, data citations are formatted as follows: "Data ref: Smith et al, 2001" or "Data ref: NCBI Sequence Read Archive PRJNA342805, 2017". In the Reference list, data citations must be labeled with "[DATASET]". A data reference must provide the database name, accession number/identifiers and a resolvable link to the landing page from which the data can be accessed at the end of the reference. Further instructions are available at: https://link.springer.com/journal/44319/submission-guidelines#cms-Reference-guidelines

7) Regarding data quantification and statistics, please make sure that the number "n" for how many independent experiments were performed, their nature (biological versus technical replicates), the bars and error bars (e.g. SEM, SD) and the test used to calculate p-values is indicated in the respective figure legends (also for EV and Appendix figures). Please also check that all the p-values are explained in the legend, and that these fit to those shown in the figure. Please provide statistical testing where applicable. Please avoid the phrase 'independent experiment' but clearly state if these were biological or technical replicates. Please also indicate (e.g. with n.s.) if testing was performed, but the differences are not significant. In case n=2, please show the data as separate datapoints without error bars and statistics. See also:
https://link.springer.com/journal/44319/submission-guidelines#cms-Figure-and-data-presentation

If n<5, please show single datapoints for diagrams.

8) Please add scale bars of similar style and thickness to all microscopic images, using clearly visible black or white bars (depending on the background). Please place these in the lower right corner of the images themselves. Please do not write on or near the bars in the image but define the size in the respective figure legend.

9) Please also note our reference format:
https://link.springer.com/journal/44319/submission-guidelines#cms-Reference-guidelines

10) We updated our journal's competing interests policy in January 2022 and request authors to consider both actual and perceived competing interests. Please review the policy https://www.embopress.org/competing-interests and update your competing interests if necessary. Please name this section 'Disclosure and Competing Interests Statement' and put it after the Acknowledgements section.
See: https://link.springer.com/partners/embo-press/editorial-policies#Competing%20interest%20disclosures

11) We now use CRediT to specify the contributions of each author in the journal submission system. CRediT replaces the author contribution section. Please use the free text box to provide more detailed descriptions and do NOT provide your final manuscript text file with an author contributions section. See also our guide to authors (section 'Author contributions'):
https://link.springer.com/journal/44319/submission-guidelines#cms-Revised-submissions

12) All Materials and Methods need to be described in the main text using our 'Structured Methods' format, which is required for all research articles. According to this format, the Methods section should include a Reagents and Tools Table (listing key reagents, experimental models, software, and relevant equipment and including their sources and relevant identifiers), uploaded as separate file, and a Methods section in which we encourage the authors to describe their methods using a step-by-step protocol format with bullet points, to facilitate the adoption of the methodologies across labs. More information on how to adhere to this format as well as downloadable templates (.doc) for the Reagents and Tools Table can be found in our author guidelines (section 'Structured Methods'):

https://link.springer.com/journal/44319/submission-guidelines#cms-Manuscript-organisation-and-formatting

Please move all antibody or primer information to the Reagents & Tools Table.

13) Please order the manuscript sections like this, using only these names:
Title page - Abstract (max. 175 words) - Keywords (up to five) - Introduction - Results - Discussion - Methods - Data availability

section - Acknowledgements (please include here all the funding information) - Disclosure and Competing Interests Statement - References - Figure legends - Expanded View Figure legends

14) Please make sure that all the funding information is also entered into the online submission system and that it is complete and similar to the one in the acknowledgement section of the manuscript text file.

In addition, I would need from you:
- a short, two-sentence summary of the manuscript (not more than 35 words).
- three to four short (!) one sentence bullet points highlighting the key findings of your study.
- a schematic summary figure (synopsis image) in jpeg or tiff format with the exact width of 550 pixels and a height of exactly 300 pixels that can be used as a visual synopsis on our website.

I look forward to seeing a revised version of your manuscript when it is ready. Please let me know if you have questions or comments regarding the revision.

We would also welcome the submission of cover suggestions, or motifs to be used by our Graphics Illustrator in designing a cover.

As part of the EMBO publication's Transparent Editorial Process, EMBO reports publishes online a Review Process File (RPF) to accompany accepted manuscripts. This File will be published in conjunction with your paper and will include the referee reports, your point-by-point response and all pertinent correspondence relating to the manuscript.

You are able to opt out of this by letting the editorial office know (emboreports@embo.org). If you do opt out, the Review Process File link will point to the following statement: "No Review Process File is available with this article, as the authors have chosen not to make the review process public in this case."

I look forward to seeing a revised form of your manuscript when it is ready.

Yours sincerely,

Achim Breiling
Senior Editor
EMBO Reports

Referee #1:

The revised version with new experiments is much improved. In particular, the follow-up experiments are more logical and connected to the primary proteomics analysis. The presentation is also clearer. The authors are commended for their high quality study.

Referee #2:

The authors have considered reviewer concerns and made a number of significant improvements to the manuscript, including additional data and analysis that address some key unanswered questions. Additional clarification to methods that were needed have also been added. The manuscript is now suitable for publication and will be a significant contribution to the field.

Referee #3:

The authors have spent extensive time and energy to address the concerns raised in my prior review. Like R1 and R2, I believe that the proteomics dataset will be a significant resource for the field. However, like R1, I continue to think that the novelty of most findings is somewhat limited. Nevertheless, on the strength of the proteomics data I find the current study suitable for publication.

The authors have addressed the editorial requests.

Dear Dr. Ly,

Thank you for the submission of your further revised manuscript to our editorial offices.
Before we can proceed with formal acceptance, I have the editorial requests below I ask you to address in a final revised manuscript. Please also provide a final p-b-p-response to the editorial requests.

Editorial requests:

- Please provide the author names on the title page in the same format as in the submission system (First Name Last Name).

- Please provide the abstract written in present tense throughout.

- Please add up to 5 keywords to the manuscript and make sure the final manuscript text file is ordered like this, using only these names:
Title page - Abstract - Keywords - Introduction - Results - Discussion - Methods - Data availability section - Acknowledgements - Disclosure and Competing Interests Statement - References - Figure legends

- Please make sure that the number "n" for how many independent experiments were performed, their nature (biological versus technical replicates), the bars and error bars (e.g. SEM, SD) and the test used to calculate p-values is indicated in the respective figure legends (main and EV figures). Please also check that all the p-values are explained in the legend, and that this fit to those shown in the figure. Please provide statistical testing where applicable. Please avoid the phrase 'independent experiment' if possible but clearly state if these were biological or technical replicates. Please also indicate (e.g. with n.s.) if testing was performed, but the differences are not significant. In case n=2, please show the data as separate datapoints without error bars and statistics. See also:

https://link.springer.com/journal/44319/submission-guidelines#cms-Figure-and-data-presentation

Presently, many diagrams have no statistics (e.g. those in Figs. 1b, 3, 4, 6b/c, 7b, 8, EV1, EV2 and EV4h), have only partial statistics (e.g. panels 5f, 5i or 6d) or might lack the 'n.s.'. Please check and provide complete statistics for all diagrams shown (with n>2). Moreover:
- Please define the annotated p values ****/***/**/* as well as provide the exact p-values for the same in the legend of figure EV3 F as appropriate.
- Please note that the exact p values are not provided in the legends of figures 1D, 7D, F; EV4 B, G.
- Please indicate the statistical test used for data analysis in the legends of figures 5B, EV3 B, F
- Please note that information related to n is missing in the legends of figures 5B, F, H, I; 8D-H
- Please note that the error bars are not defined in the legends of figures 1B, D; 5B, F, I; 7B, D, E, F, G, H; EV3 B, F

- For panel 1b n seems to be 2. Please show the two independent experiments separated.

- Please make sure that each figure panel is called out separately and that the panels are called out sequentially. Moreover, the correct nomenclature for the EV Figures is 'Figure EVx'. Please use this format for their callouts (not 'Fig. Sx').

- There are four Supplementary Tables uploaded. These are datasets. Please name and upload these as 'Dataset EV1 - Dataset EV4' ("Supplementary" should not be used) and update their callouts. Please make sure they have a legend on the first TAB of the excel file.

- Please use our reference format:
https://link.springer.com/journal/44319/submission-guidelines#cms-Reference-guidelines

Moreover, et al needs to be used after 10 author names.

- Please add nucleic acid (primer, gRNA) information still present in the Methods section directly to the Reagents and Tools Table and remove these from the Methods. Please add callouts ('see Reagents & Tools table') to the Reagents & Tools Table were appropriate. Please also remove the instructions and samples sections from the final Reagents & Tools Table.

- Please check again that numerical source data (SD) is provided for all diagrams shown (e.g. for those in Figs. 2, 3, 4, 6, 7b, 8, EV1, EV2a/b, EV3b/c/e - for all these SD is presently lacking). In case, please update the source data checklist.

- Please remove now the referee token form the data availability section and add a direct link to the deposited dataset. Please make sure the dataset is public latest upon online publication of the study.

- Please provide a schematic summary figure as separate file that provides a sketch of the major findings (not a data image) in

jpeg or tiff format (with the exact width of 550 pixels and the exact height of 300 pixels) that can be used as a visual synopsis on our website.

I look forward to seeing the further revised version of your manuscript when it is ready. Please let me know if you have questions regarding the revision.

Best,

Achim Breiling
Senior Editor
EMBO Reports

The authors have addressed the additional editorial requests.

Tony Ly
University of Dundee
School of Life Sciences
United Kingdom

Dear Dr. Ly,

I am very pleased to accept your manuscript for publication in the next available issue of EMBO reports. Thank you for your contribution to our journal.

Your manuscript will be processed for publication by EMBO Press. It will be copy edited and you will receive page proofs prior to publication. Please note that you will be contacted by Springer Nature Author Services to complete licensing and payment information.

You may qualify for financial assistance for your publication charges - either via a Springer Nature fully open access agreement or an EMBO initiative. Check your eligibility: https://link.springer.com/journal/44319/how-to-publish-with-us

Should you be planning a Press Release on your article, please notify the production team via this form in order to coordinate publication and release dates: https://forms.cloud.microsoft/e/eH4A8JVP6g

If you have any questions, please do not hesitate to contact the Editorial Office. Thank you for your contribution to EMBO Reports.

Yours sincerely,

Achim Breiling
Senior Editor
EMBO Reports

>>> Please note that it is EMBO Reports policy for the transcript of the editorial process (containing referee reports and your response letter) to be published as an online supplement to each paper. If you do NOT want this, you will need to inform the Editorial Office via email immediately. More information is available here: https://link.springer.com/partners/embo-press/editorial-policies#Peer%20review

***
Rev_Com_number: RC-2025-02944
New_manu_number: EMBOR-2026-63971V3
Corr_author: Ly
Title: CD8 T lymphocytes deploy embryonic cell cycle control mechanisms for rapid cell proliferation

EMBO Press Author Checklist

Corresponding Author Name: Tony Ly	
Journal Submitted to: EMBO Reports	
Manuscript Number: EMBOR-2026-63971V1-T	

USEFUL LINKS FOR COMPLETING THIS FORM
The EMBO Journal - Author Guidelines
EMBO Reports - Author Guidelines
Molecular Systems Biology - Author Guidelines
EMBO Molecular Medicine - Author Guidelines

Reporting Checklist for Life Science Articles (updated January
This checklist is adapted from Materials Design Analysis Reporting (MDAR) Checklist for Authors. MDAR establishes a minimum set of requirements in transparent reporting in the life sciences (see Statement of Task: 10.31222/osf.io/9sm4x). Please follow the journal's guidelines in preparing your
Please note that a copy of this checklist will be published alongside your article.

Abridged guidelines for figures
1. Data
The data shown in figures should satisfy the following conditions:
- the data were obtained and processed according to the field's best practice and are presented to reflect the results of the experiments in an accurate and unbiased manner.
- ideally, figure panels should include only measurements that are directly comparable to each other and obtained with the same assay.
- plots include clearly labeled error bars for independent experiments and sample sizes. Unless justified, error bars should not be shown for technical
- if n<5, the individual data points from each experiment should be plotted. Any statistical test employed should be justified.
- Source Data should be included to report the data underlying figures according to the guidelines set out in the authorship guidelines on Data

2. Captions
Each figure caption should contain the following information, for each panel where they are relevant:
- a specification of the experimental system investigated (eg cell line, species name).
- the assay(s) and method(s) used to carry out the reported observations and measurements.
- an explicit mention of the biological and chemical entity(ies) that are being measured.
- an explicit mention of the biological and chemical entity(ies) that are altered/varied/perturbed in a controlled manner.
- the exact sample size (n) for each experimental group/condition, given as a number, not a range;
- a description of the sample collection allowing the reader to understand whether the samples represent technical or biological replicates (including how many animals, litters, cultures, etc.).
- a statement of how many times the experiment shown was independently replicated in the laboratory.
- definitions of statistical methods and measures:
  - common tests, such as t-test (please specify whether paired vs. unpaired), simple χ2 tests, Wilcoxon and Mann-Whitney tests, can be unambiguously identified by name only, but more complex techniques should be described in the methods section;
  - are tests one-sided or two-sided?
  - are there adjustments for multiple comparisons?
  - exact statistical test results, e.g., P values = x but not P values < x;
  - definition of 'center values' as median or average;
  - definition of error bars as s.d. or s.e.m.

Please complete ALL of the questions below.
Select "Not Applicable" only when the requested information is not relevant for your study.

Materials

Newly Created Materials	Information included in the manuscript?	In which section is the information available? (Reagents and Tools Table, Materials and Methods, Figures, Data Availability Section)
New materials and reagents need to be available; do any restrictions apply?	Not Applicable	

Antibodies	Information included in the manuscript?	In which section is the information available? (Reagents and Tools Table, Materials and Methods, Figures, Data Availability Section)
For **antibodies** provide the following information:<br>- Commercial antibodies: RRID (if possible) or supplier name, catalogue number and or/clone number<br>- Non-commercial: RRID or citation	Yes	Reagents and Tools Table

DNA and RNA sequences	Information included in the manuscript?	In which section is the information available? (Reagents and Tools Table, Materials and Methods, Figures, Data Availability Section)
**Short novel DNA or RNA including primers, probes**: provide the sequences.	Yes	Reagents and Tools Table

Cell materials	Information included in the manuscript?	In which section is the information available? (Reagents and Tools Table, Materials and Methods, Figures, Data Availability Section)
**Cell lines:** Provide species information, strain. Provide accession number in repository **OR** supplier name, catalog number, clone number, and/**OR** RRID.	Yes	Reagents and Tools Table
**Primary cultures:** Provide species, strain, sex of origin, genetic modification status.	Not Applicable	Methods and Protocols section
Report if the cell lines were recently **authenticated** (e.g., by STR profiling) and tested for mycoplasma contamination.	Not Applicable	

Experimental animals	Information included in the manuscript?	In which section is the information available? (Reagents and Tools Table, Materials and Methods, Figures, Data Availability Section)
**Laboratory animals or Model organisms:** Provide species, strain, sex, age, genetic modification status. Provide accession number in repository **OR** supplier name, catalog number, clone number, **OR** RRID.	Yes	Methods and Protocols section
**Animal observed in or captured from the field:** Provide species, sex, and age where possible.	Not Applicable	
Please detail **housing and husbandry conditions**.	Yes	Methods and Protocols section

Plants and microbes	Information included in the manuscript?	In which section is the information available? (Reagents and Tools Table, Materials and Methods, Figures, Data Availability Section)
**Plants:** provide species and strain, ecotype and cultivar where relevant, unique accession number if available, and source (including location for collected wild specimens).	Not Applicable	
**Microbes:** provide species and strain, unique accession number if available, and source.	Not Applicable	

Human research participants	Information included in the manuscript?	In which section is the information available? (Reagents and Tools Table, Materials and Methods, Figures, Data Availability Section)
If collected and within the bounds of privacy constraints report on age, sex and gender or ethnicity for all study participants.	Not Applicable	

Core facilities	Information included in the manuscript?	In which section is the information available? (Reagents and Tools Table, Materials and Methods, Figures, Data Availability Section)
If your work benefited from core facilities, was their service mentioned in the acknowledgments section?	Yes	

Design

Study protocol	Information included in the manuscript?	In which section is the information available? (Reagents and Tools Table, Materials and Methods, Figures, Data Availability Section)
If study protocol has been **pre-registered, provide DOI in the manuscript**. For clinical trials, provide the trial registration number **OR** cite DOI.	Not Applicable	
Report the **clinical trial registration number** (at ClinicalTrials.gov or equivalent), where applicable.	Not Applicable	

Laboratory protocol	Information included in the manuscript?	In which section is the information available? (Reagents and Tools Table, Materials and Methods, Figures, Data Availability Section)
Provide DOI OR other citation details if **external detailed step-by-step protocols** are available.	Not Applicable	

Experimental study design and statistics	Information included in the manuscript?	In which section is the information available? (Reagents and Tools Table, Materials and Methods, Figures, Data Availability Section)
Include a statement about **sample size** estimate even if no statistical methods were used.	Yes	Methods and Protocols and Figure captions
Were any steps taken to minimize the effects of subjective bias when allocating animals/samples to treatment (e.g. **randomization procedure**)? If yes, have they been described?	Not Applicable	
Include a statement about **blinding** even if no blinding was done.	Not Applicable	
Describe **inclusion/exclusion criteria** if samples or animals were excluded from the analysis. Were the criteria pre-established?<br><br>If sample or data points were omitted from analysis, report if this was due to attrition or intentional exclusion and provide justification.	Not Applicable	
For every figure, are **statistical tests** justified as appropriate? Do the data meet the assumptions of the tests (e.g., normal distribution)? Describe any methods used to assess it. Is there an estimate of variation within each group of data? Is the variance similar between the groups that are being statistically compared?	Yes	Methods and Protocols

Sample definition and in-laboratory replication	Information included in the manuscript?	In which section is the information available? (Reagents and Tools Table, Materials and Methods, Figures, Data Availability Section)
In the figure legends: state number of times the experiment was **replicated** in laboratory.	Yes	
In the figure legends: define whether data describe **technical or biological replicates**.	Yes	

Ethics

Ethics	Information included in the manuscript?	In which section is the information available? (Reagents and Tools Table, Materials and Methods, Figures, Data Availability Section)
Studies involving **human participants**: State details of **authority granting ethics approval** (IRB or equivalent committee(s), provide reference number for approval.	Not Applicable	
Studies involving **human participants**: Include a statement confirming that **informed consent** was obtained from all subjects and that the experiments conformed to the principles set out in the WMA Declaration of Helsinki and the Department of Health and Human Services Belmont Report.	Not Applicable	
Studies involving **human participants:** For publication of **patient photos**, include a statement confirming that consent to publish was obtained.	Not Applicable	
Studies involving experimental **animals**: State details of **authority granting ethics approval** (IRB or equivalent committee(s), provide reference number for approval. Include a statement of compliance with ethical regulations.	Yes	Methods and Protocols
Studies involving **specimen and field samples:** State if relevant **permits** obtained, provide details of authority approving study; if none were required, explain why.	Not Applicable	

Dual Use Research of Concern (DURC)	Information included in the manuscript?	In which section is the information available? (Reagents and Tools Table, Materials and Methods, Figures, Data Availability Section)
Could your study fall under dual use research restrictions? Please check biosecurity documents and list of **select agents and toxins** (CDC): https://www.selectagents.gov/sat/list.htm	Not Applicable	
If you used a select agent, is the security level of the lab appropriate and reported in the manuscript?	Not Applicable	
If a study is subject to dual use research of concern regulations, is the name of the **authority granting approval and reference number** for the regulatory approval provided in the manuscript?	Not Applicable	

Reporting

The MDAR framework recommends adoption of discipline-specific guidelines, established and endorsed through community initiatives. Journals have their own policy about requiring specific guidelines and recommendations to complement MDAR.

Adherence to community standards	Information included in the manuscript?	In which section is the information available? (Reagents and Tools Table, Materials and Methods, Figures, Data Availability Section)
State if relevant guidelines or checklists (e.g., **ICMJE, MIBBI, ARRIVE, PRISMA**) have been followed or provided.	Not Applicable	
For **tumor marker prognostic studies,** we recommend that you follow the **REMARK** reporting guidelines (see link list at top right). See author guidelines, under 'Reporting Guidelines'. Please confirm you have followed these guidelines.	Not Applicable	
For **phase II and III randomized controlled trials**, please refer to the **CONSORT** flow diagram (see link list at top right) and submit the CONSORT checklist (see link list at top right) with your submission. See author guidelines, under 'Reporting Guidelines'. Please confirm you have submitted this list.	Not Applicable	

Data Availability

Data availability	Information included in the manuscript?	In which section is the information available? (Reagents and Tools Table, Materials and Methods, Figures, Data Availability Section)
Have **primary datasets** been deposited according to the journal's guidelines (see 'Data Deposition' section) and the respective accession numbers provided in the Data Availability Section?	Yes	Data Availability section
Were **human clinical and genomic datasets** deposited in a public access-controlled repository in accordance to ethical obligations to the patients and to the applicable consent agreement?	Not Applicable	
Are **computational models** that are central and integral to a study available without restrictions in a machine-readable form? Were the relevant accession numbers or links provided?	Not Applicable	
If publicly available data were reused, provide the respective **data citations in the reference list**.	Yes	
