# Supplementary material for: CD8 T lymphocytes deploy embryonic cell cycle control mechanisms for rapid cell proliferation
Source: EMBO Rep. 2026 Jun 18;27(15):4272–98. doi: 10.1038/s44319-026-00830-4 (PMC13458459; doi:10.1038/s44319-026-00830-4)
Supplement: Supplementary file 5 [file 44319_2026_830_MOESM6_ESM.zip › Figure 1/1c/mESC/EdU-staining-test/12-Jun-2024-Layout.pdf]

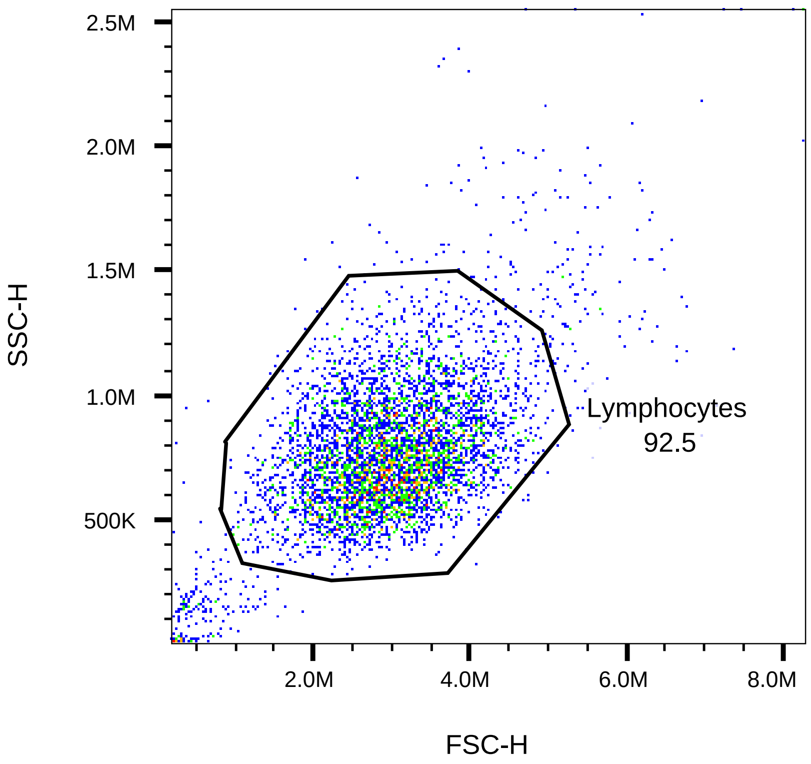

A1_mESC-1.fcs
Ungated
5148

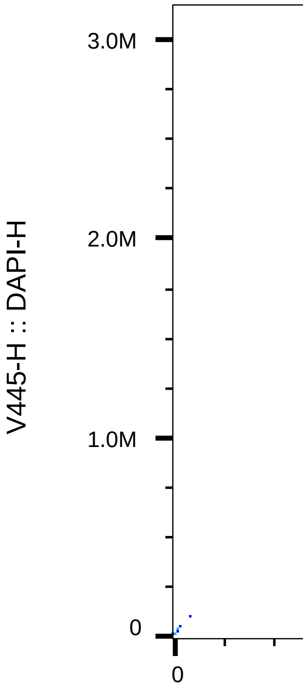

A1_mESC
Lymphocy
4764

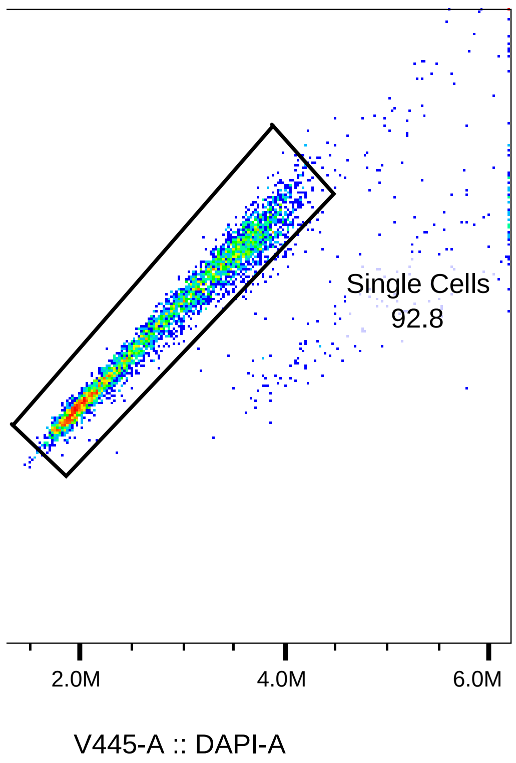

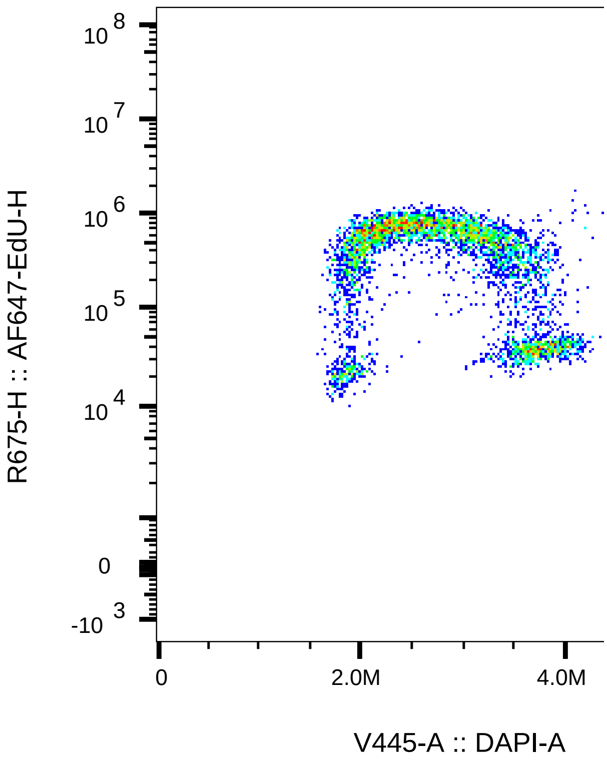

:-1.fcs
/tes

A1_mESC-1.fcs
Single Cells
4421

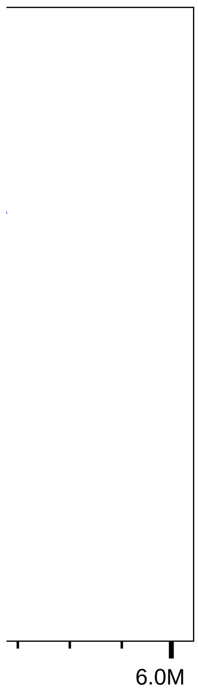
6.0M
